# Toll-like receptor-9 stimulated plasmacytoid dendritic cell precursors suppress autoimmune neuroinflammation in a murine model of multiple sclerosis

**DOI:** 10.1038/s41598-021-84023-0

**Published:** 2021-02-26

**Authors:** Hélène Letscher, Viviane A. Agbogan, Sarantis Korniotis, Pauline Gastineau, Emmanuel Tejerina, Christophe Gras, Jérôme Mégret, Alison Moe, William R. Drobyski, Flora Zavala

**Affiliations:** 1 Institut Necker Enfants Malades, INSERM U1151, CNRS UMR8253, Université de Paris, 156 rue de Vaugirard, 75015 Paris, France; 2 Structure Fédérative de Recherche Necker, INSERM US24, CNRS UMS 3633, 75015 Paris, France; 3 Department of Medicine, Medical College of Wisconsin, 8701 W Watertown Plank Rd, Milwaukee, WI 53226 USA; 4 Present Address: Institut de Recherche Saint Louis (IRSL), INSERM U976, 75010 Paris, France

**Keywords:** Immunology, Neuroscience, Stem cells

## Abstract

Early innate education of hematopoietic progenitors within the bone marrow (BM) stably primes them for either trained immunity or instead immunoregulatory functions. We herein demonstrate that in vivo or in vitro activation within the BM via Toll-like receptor-9 generates a population of plasmacytoid dendritic cell (pDC) precursors (CpG-pre-pDCs) that, unlike pDC precursors isolated from PBS-incubated BM (PBS-pre-pDCs), are endowed with the capacity to halt progression of ongoing experimental autoimmune encephalomyelitis. CpG activation enhances the selective migration of pDC precursors to the inflamed spinal cord, induces their immediate production of TGF-β, and after migration, of enhanced levels of IL-27. CpG-pre-pDC derived TGF-β and IL-27 ensure protection at early and late phases of the disease, respectively. Spinal cords of CpG-pre-pDC-protected recipient mice display enhanced percentages of host-derived pDCs expressing TGF-β as well as an accumulation of IL-10 producing B cells and of CD11c^+^ CD11b^+^ dendritic cells. These results reveal that pDC precursors are conferred stable therapeutic properties by early innate activation within the BM. They further extend to the pDC lineage promising perspectives for cell therapy of autoimmune diseases with innate activated hematopoietic precursor cells.

## Introduction

Adaptive hematopoiesis has been described as a key response to infections or injury. Indeed, hematopoietic progenitors are not only confined to the bone marrow (BM) to replenish the immune system by differentiation but can also sense pathogens and danger signals and travel to injured or inflammatory tissues^1^. Recent findings have highlighted that the innate ability of hematopoietic stem cells and progenitors to sense, respond to stress and adapt to subsequent immune responses shapes trained immunity^2,3^. Trained immunity was shown to foster expansion of myeloid progenitors driving myelopoiesis, thereby enabling a sustained response to a secondary microbial challenge^4^.

A challenging hypothesis is that primary innate education of hematopoietic progenitors may not only enhance secondary responses to infections but also induce immune regulatory responses. Askenase et al. showed that gastrointestinal infection primes monocyte precursors prior to BM egress for regulatory function within peripheral tissues^5^. Moreover, beyond myeloid progenitors, innate activated hematopoietic progenitors including multipotent^6–8^ and B-cell^9,10^ progenitors were also described to acquire regulatory properties against adaptive immune responses such as autoimmune diseases. Thus, we have shown that G-CSF, a response factor to infections, mobilizes multipotent progenitors from the bone marrow to the spleen, a process conferring to the progenitors the capacity to enhance the expansion of functional Tregs and thereby, upon adoptive transfer, protecting against type 1 diabetes (T1D) onset in Non Obese Diabetic (NOD) mice^6,7^ and experimental autoimmune encephalomyelitis, a murine model of multiple sclerosis^8^. We also showed that activation with the TLR-9 agonist CpG within the BM conferred to B-cell committed progenitors, at the pro-B cell stage (CpG-proBs), the capacity to control effector T cells and provide protection against disease in both T1D and multiple sclerosis (MS) models, at the onset of clinical signs^9^. CpG-proBs have the capacity to migrate towards the immune reaction site and target tissues, where they differentiate into diverse mature Breg subsets that act locally^9^. Altogether, these observations suggested that early education of hematopoietic progenitors within the BM may be essential to imprint regulatory functions that enable them to control immune responses in the periphery.

Optimal treatments for autoimmune conditions require efficacy when symptoms are detected, i.e. at the effector phase of disease. Cell therapy with Tregs is poorly efficient at the effector phase of EAE as Tregs tend to lose their immunosuppressive properties in an inflammatory environment^11^. Indeed, the timing and stimuli sensed by immune cells affect their capacity to restore tolerance or instead contribute to disease severity.

Similar versatility has been reported for plasmacytoid dendritic cells (pDCs). In MS patients, pDCs accumulate in the central nervous system (CNS) and the cerebrospinal fluid^12^. In the peripheral blood, although pDCs remain stable in numbers, they display a dysfunctional phenotype^13^. Interestingly, the recovery of pDC functions correlates with clinical improvement in MS patients treated with glatiramer acetate, suggesting a capacity of human pDCs to gain a regulatory role in MS.

In mice, CNS pDCs, although far less efficiently than mDCs, are able to present auto-antigen and induce a CD4^+^ T-cell response, mostly Th1- and Th17-oriented and thereby exert inflammatory influence^14^. As a result, pDC depletion before EAE induction is clinically beneficial^15^. It promotes significant reduction of circulating IFN-α and lower frequency of peripheral Th17 cells, effects that are only partially reproduced by type I IFN neutralization in pDC-replete animals^16^. These results suggested that pDCs are required for EAE initiation, in part via their IFN-I production. Conversely, pDC depletion one week after immunization results in aggravation of EAE, suggesting that pDCs adopt a regulatory function once disease has started. They inhibit CNS pro-inflammatory myeloid DCs and thereby reduce pathogenic CNS Th17 differentiation^14^. Moreover, after EAE induction, pDCs establish MHC-II dependent myelin-Ag specific contacts with CD4^+^ T-cells in lymph nodes that promote selective expansion of myelin antigen specific regulatory T cells^17^. Such property was shown to strictly depend on their pre-loading with myelin antigen^18^ whereas simple activation with CpG, although enhancing MHC-II expression, did not confer to adoptively transferred mature pDCs a protective function against EAE^18^.

Whether immune regulatory function can be stably imprinted at the early BM precursor stage and downstream the pDC lineage remains unknown.

We herein investigated whether early education of pDCs with TLR agonists during development, at the stage of pDC precursors prior to bone marrow egress, determines whether they exert pro-inflammatory or immunoregulatory functions in the context of an experimental model of multiple sclerosis, experimental autoimmune encephalomyelitis (EAE). We report that either in vivo or in vitro activation with the TLR-9 agonist CpG confers them a highly stable capacity to control ongoing EAE. CpG activation enhances the selective migration of pDC precursors to the inflamed spinal cord, induces their production of the anti-inflammatory cytokines TGF-β and, after migration, IL-27, acting sequentially for protection against disease. These results reveal that early innate activation of pDC precursors within the BM allows them to effectively control tissue-specific immunity.

## Results

### Emergence of pDC precursors in bone marrow after CpG activation: isolation, phenotypic and functional characterization

**Figure 1:**
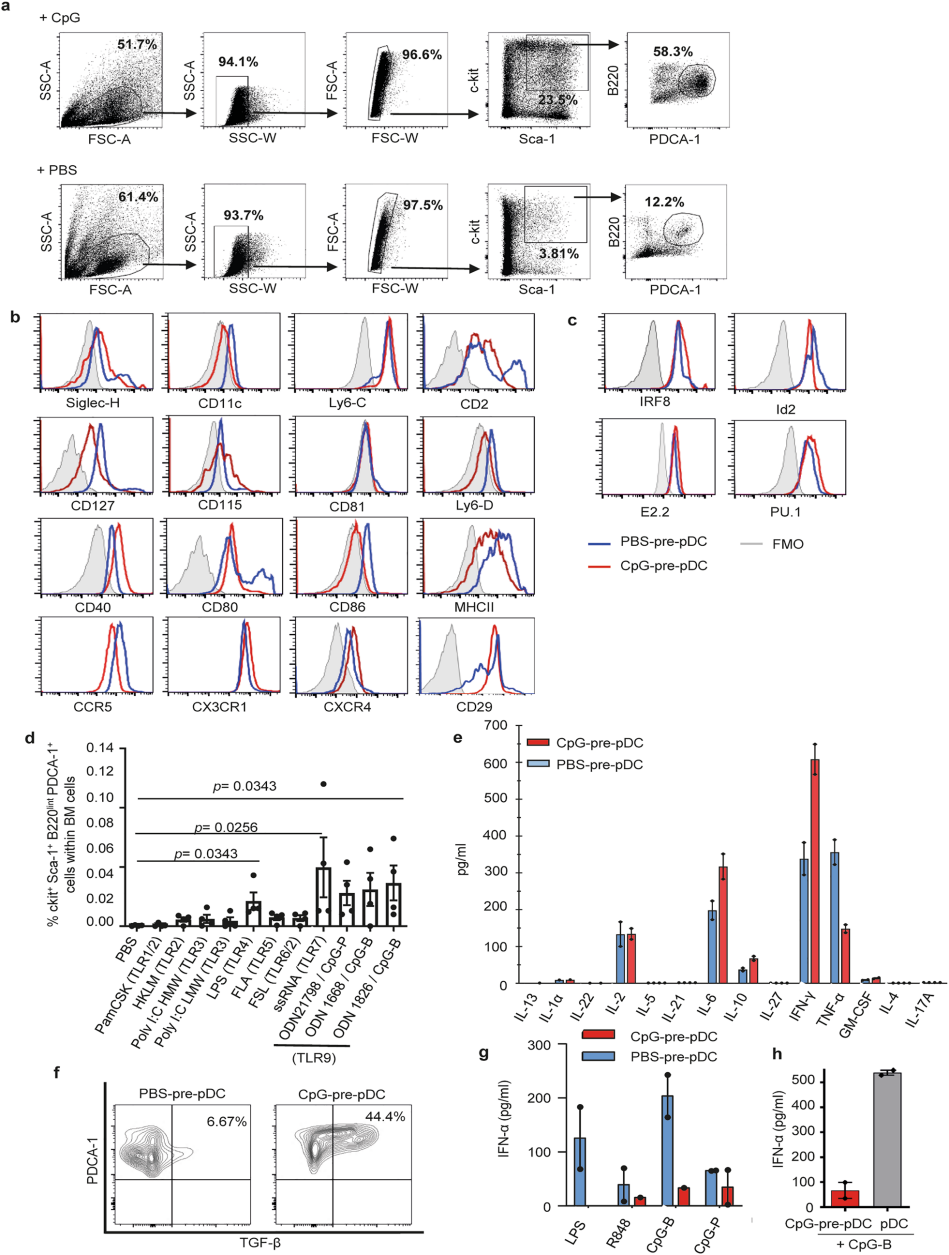
Cell sorting strategy, phenotypic and functional characterization of CpG-induced c-kit^+^Sca-1^+^B220^int^PDCA-1^+^ BM cells. (**a**) Total BM cells incubated with PBS or CpG-B (1 µg/ml) for 18 h and magnetically enriched for c-kit^+^ cells were further labelled for Sca-1, B220, PDCA-1 and electronically sorted into c-kit^+^Sca-1^+^B220^int^PDCA-1^+^ cells. Flow cytometry dot plots representative of 30 experiments. (**b**) Further characterization of cell-sorted CpG- as well as PBS-induced BM population was performed using flow cytometry analysis of the expression of various myeloid and dendritic cell markers as well as cell migration receptors. Specific antibody staining is depicted with open histograms (blue line for PBS- and red line for CpG-induced cells). Positive cells are defined using FMO controls (grey histograms). Two experiments. (**c**) FACS analysis of intranuclear expression of transcription factors. Analysis of IRF8 and Id2 expression with specific antibodies compared to FMO controls and of E2.2 and PU.1 by flowRNA, compared with B220^+^PDCA-1^−^ control B cells (grey histograms), in CpG- versus PBS-stimulated c-kit^+^Sca-1^+^B220^int^PDCA-1^+^ population. Two experiments. (**d**) Frequency of c-kit^+^Sca-1^+^B220^int^PDCA-1^+^ cells emerging among total BM cells after 18 h of incubation with different TLR agonists, measured in BM cell cultures of four individual mice. Statistical significance as indicated, analyzed by Mann–Whitney test. (**e**) Cytokine production of cell-sorted PBS- versus CpG-induced c-kit^+^Sca-1^+^B220^int^PDCA-1^+^ cells, measured in the supernatant after 4 h of activation with PMA/ionomycin in the presence of brefeldin A using multiplex ELISA. Biological duplicates. (**f**) Intracytoplasmic TGF-β expression was analyzed by FACS in PBS- and CpG-pre-pDCs. One experiment out of two (**g**) IFN-α production was measured by ELISA in supernatants of CpG- versus PBS-induced cell-sorted c-kit^+^Sca-1^+^B220^int^PDCA-1^+^ BM cells after 18 h incubation with 1 μg/ml TLR-7 (R848), TLR-9 (CpG-B and CpG-P) and TLR-4 (LPS) agonists (biological duplicates). (**h**) Mature spleen pDCs and CpG-pre-pDCs were compared for their IFN-α response after 18-h incubation with CpG-B (1 μg/ml), measured by ELISA in supernatants (biological duplicates).

Incubation of a BM cell suspension with CpG-B (10 µg/ml) for 18 h led to the accumulation of hematopoietic progenitors that were first enriched by c-kit positive magnetic selection. Upon further staining, we proceeded to FACS-cell sorting by elimination of doublets followed by gating of c-kit^+^Sca-1^+^ cells, then B220^int^PDCA-1^+^ cells. We identified a fraction expressing the phenotype c-kit^+^Sca-1^+^B220^+^PDCA-1^+^ (Fig. 1a). This PDCA-1^+^ cell population expressed only low levels of the myeloid lineage marker CD115 (M-CSFR) but expressed CD2, CD127 (IL7-Rα) and low levels of Ly6D. Altogether, its c-kit^+^Sca-1^+^B220^low^PDCA-1^+^CD11c^low^Siglec-H^+^Ly6C^+^Ly6D^low^CD2^+^CD127^+^CD115^−^ phenotype (Fig. 1b) suggested a plasmacytoid dendritic cell precursor derived from the common lymphoid progenitor (CLP)^19–21^ that we named CpG-pre-pDCs. Notably, comparison with a c-kit^+^Sca-1^+^B220^+^PDCA-1^+^ fraction sorted from the BM with 25-fold lower frequency among c-kit^+^ cells after 18 h incubation with PBS instead (PBS-pre-pDCs) showed that CpG activation reduced CD2 and Ly6D expression levels. It also reduced MHC II, CD80 and CD86 but enhanced CD40 expression levels. Additionally, CpG modulated the expression of various receptors playing a role in migration^22–24^, reducing CCR5 expression but enhancing CX3CR1, CXCR4 and notably CD29 expression (Fig. 1b), the latter reported to condition pDC migration to the CNS during neuroinflammation^25^.

Engagement of the CpG-activated cell-sorted progenitor population into the pDC lineage was further confirmed by assessing the respective nuclear expression of several transcription factors including the basic helix–loop–helix transcription factor E2-2 (also known as TCF4) which is essential for the specification, commitment as well as maintenance of the pDC identity^26,27^ and Id2, a repressor of E2.2 that drives cDC development instead^28^. Additionally, we checked for IRF8, which is activated by E2.2^29^ as well as PU.1, the latter active in early lympho-myeloid development and reported to be expressed by pDC precursors^30^. Comparison with PBS-pre-pDCs (Fig. 1a) showed that activation with CpG has nearly no effect on the expression level of both E2-2 and PU.1 in the sorted population (Fig. 1c) whereas expression of IRF8 was enhanced and conversely Id2 reduced. The observed transcription factors expression confirms the pDC lineage of the BM precursors.

A survey of various TLR agonists demonstrated that only TLR-4, TLR-7 and TLR-9 agonists were able to promote the emergence of this population within BM cell culture at significant levels (Fig. 1d).

The functional properties of CpG-pre-pDCs and PBS-pre-pDCs were examined. After activation with PMA + ionomycin, both PBS- and CpG-induced progenitors released essentially IFN-γ, IL-6, IL-2, TNF-α and IL-10, fitting with a typical cytokine profile of pDCs, with no major differences between the two emergence conditions, except for enhanced IFN-γ and reduced TNF-α upon CpG activation (Fig. 1e). No significant IL-27 production was measured in any condition. Notably, only CpG-activated pDC precursors expressed TGF-β (Fig. 1f). Furthermore, as previously reported for human^31^ and murine Sca-1^+^ BM pDC progenitors^32^, they showed restricted ability, nearly abrogated for CpG-pre-pDCs, to produce IFN-α in response to activation by the TLR-9 agonists CpG-B and CpG-P (a form of the oligonucleotide specific for activating pDCs) as well as by the TLR-7 agonist R848 and the TLR-4 agonist LPS (Fig. 1g). CpG-pre-pDCs produced nearly tenfold less IFN-α than mature spleen pDCs in response to CpG-B stimulation (Fig. 1h).

Altogether, these key characteristics support the identification of the cell fraction isolated from CpG-incubated BM suspension displaying the following phenotype: c-kit^+^Sca-1^+^CD115^−^CD11c^low^Siglec H^+^PDCA-1^+^Ly6C^+^Ly6D^+^CD2^+^CD115^−^E2.2^+^IRF8^+^ Id2^+^PU-1^hi^, standing for a pDC precursor.

### CpG-pre-pDCs provide protection against EAE at the onset of clinical signs

**Figure 2:**
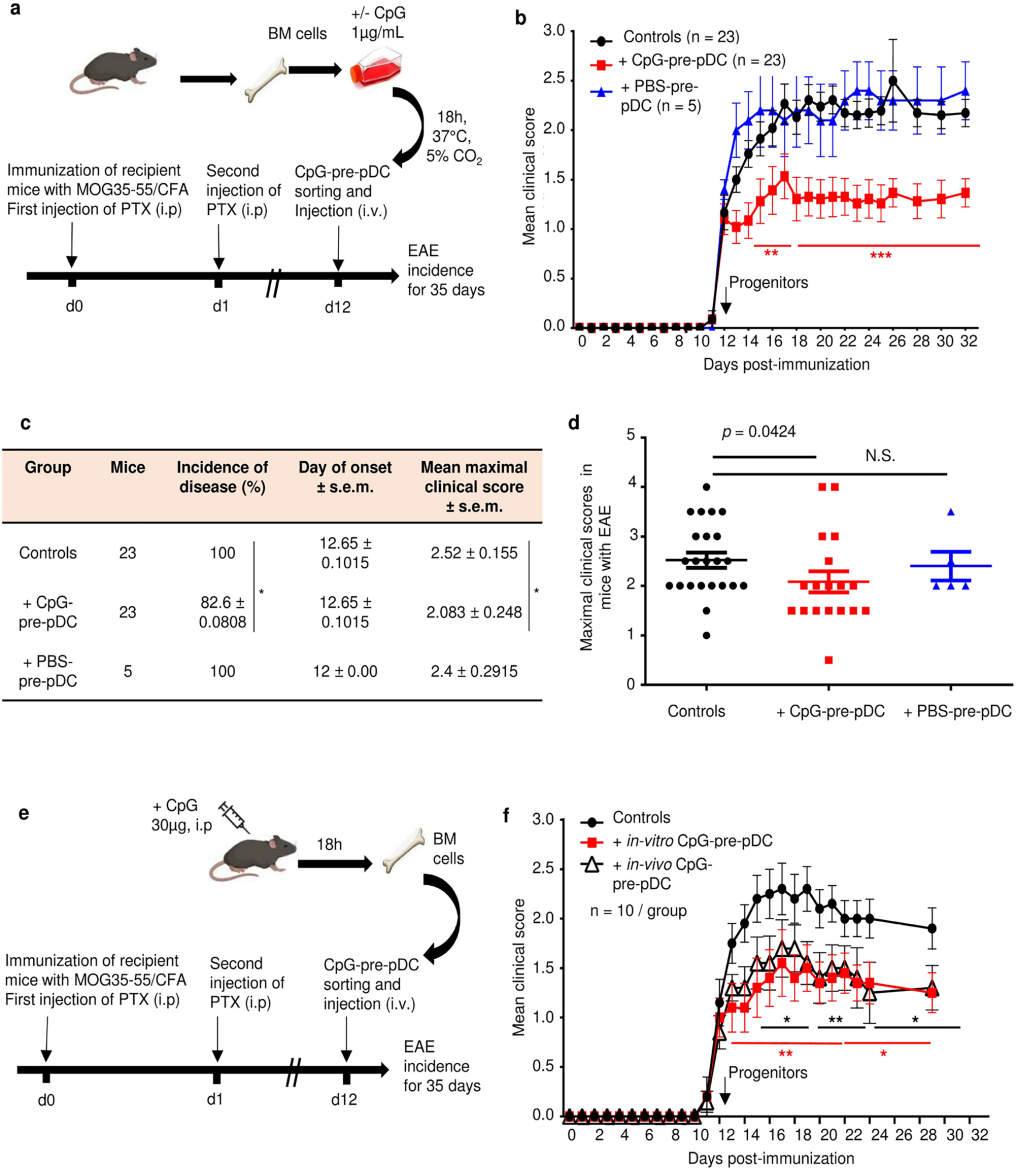
EAE disease protection by pDC progenitors. c-kit^+^Sca-1^+^B220^int^PDCA-1^+^ cells were cell-sorted as in Fig. 1a from (**a**) BM cells stimulated in vitro for 18 h with CpG-B (1 µg/ml) or PBS, and 80,000 cells injected iv into MOG_35–55_-immunized mice at the onset of EAE clinical signs (d-12)**.** Controls are mice immunized for EAE but injected with PBS medium only. (**b**) EAE clinical scores (mean ± s.e.m.) were measured over 35 days for MOG_35-55_ immunized C57Bl/6 J mice and injected with CpG-pre-pDCs (n = 23 mice per group) or PBS-pre-pDCs (n = 5 mice) or PBS (controls, n = 23 mice), data obtained from three cumulated experiments. Statistical analysis was performed using two-way ANOVA with Bonferroni post-test, d14–d17, **, *p* < 0.004, d18–d30 ****, *p* = 0.0003 when comparing CpG-pre-pDC injected mice with controls (**c**, **d**) Incidence of disease, and mean clinical scores in mice with EAE, but not day of onset were significantly modified by CpG-pre-pDCs but not by PBS-pre-pDCs, *, *p* = 0.0458, for incidence of disease, by Log-rank test and *, *p* = 0.0424, for maximal clinical score between controls vs CpG-pre-pDC recipients, by Mann–Whitney test. (**e**, **f**) BM cells isolated from mice 18 h after in vivo injection of CpG-B (30 μg/mouse, i.p.) were compared with in vitro-induced CpG-pre-pDCs for their effect on EAE upon injection at d-12 (n = 10 mice/group). Two cumulated experiments. Statistical analysis was performed using two-way ANOVA with Bonferroni post-test, Controls vs in vitro prepared CpG-pre-pDCs: d13–d21 **, *p* < 0.004, d23–d29 *, *p* = 0.0177; Controls vs in vivo prepared CpG-pre-pDCs: d14–d17 *, *p* < 0.032, d19–d21**, *p* < 0.0018, d23–d29, *, *p* < 0.047. Mice in (**a**) and (**e**) were created with Biorender.com.

To investigate any possible protective properties in an autoimmune disease, the CpG-pre-pDC progenitors were adoptively transferred in mice immunized for experimental autoimmune encephalomyelitis (EAE), a model of multiple sclerosis (Fig. 2a,b). A single intravenous injection of only 80,000 CpG-pre-pDCs per recipient at the onset of clinical signs (day-12) significantly protected against EAE (Fig. 2b). CpG stimulation was absolutely required for the protective effect of PDCA-1^+^ precursor population since the cell fraction recovered after incubation of BM cells with PBS, instead of CpG, conferred no protection (Fig. 2b). Both disease incidence (Fig. 2c) and severity assessed by maximum clinical scores in mice with EAE (Fig. 2c,d) were reduced by CpG-pre-pDCs. Moreover, the same PDCA-1^+^ precursor fraction cell-sorted from the BM of mice 18 h after an i.p. injection of CpG-B (30 µg/recipient) (Supplementary Fig. S1), representing 1.37% of total BM cells (enriched approximately tenfold over pre-pDCs that represent 0.1% of total cells in PBS-injected mice) and 0.98% of c-kit^+^ BM cells, respectively, exerted a protective effect against EAE similar to that achieved by in vitro activated CpG-pre-pDCs (Fig. 2e,f), suggesting the pathophysiological relevance of this precursor cell population.

### Fate and functional properties of adoptively transferred CpG-pre-pDCs in EAE-developing recipients

**Figure 3:**
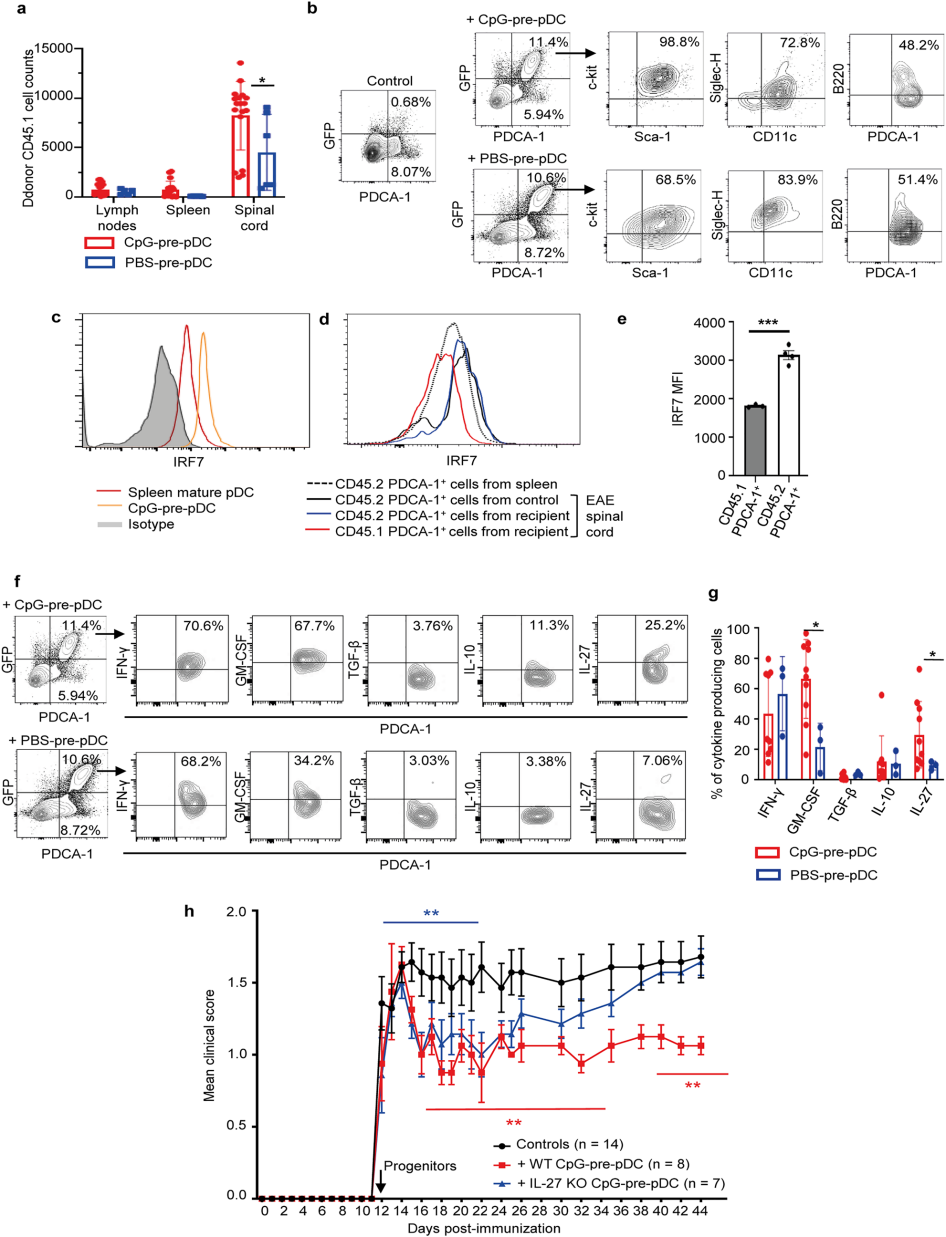
Fate and functional properties of CpG-pre-pDCs in EAE mice. CD45.1 (**a**) or GFP^+^ (**b**) CpG-pre-pDCs were injected at d-12 into MOG_35–55_ immunized CD45.2 mice. (**a**) Absolute numbers of the CD45.1 cells were analyzed in the spleen, draining lymph nodes and spinal cord of the animals at d-18 (mean ± s.e.m.), n = 17 mice with CpG-pre-pDCs, n = 5 mice with PBS-pre-pDCs, from two cumulated experiments, *p = 0.02, using unpaired Students’ *t*-test. (**b**) Phenotype of the spinal cord GFP^+^ cells, assessed at d-18 using flow cytometry analysis with FMO controls. One representative experiment out of 4. (**c**–**e**) FACS analysis of intranuclear IRF7 expression (**c**) in bone marrow CpG-pre-pDCs and spleen derived pDCs, (**d**) in CD45.1 CpG-pre-pDCs recovered from the spinal cord of EAE recipients, compared to host CD45.2 PDCA-1^+^ cells, either from CpG-pre-pDC recipients or from control mice with EAE injected with PBS medium. (**e**) MFI values of IRF7 were plotted for CD45.1 PDCA-1^+^ and host CD45.2 PDCA-1^+^ cells from 4 CpG-pre-pDC recipient mice with EAE. Mean ± s.e.m. Statistical analysis was performed using unpaired *t*-test. *** *p* < 0.001. (**f**) Cytokine production of the CD45.1 cells recovered from the spinal cord analyzed at d-18 by flow cytometry with isotype controls after 4 h activation with PMA/ionomycin in presence of brefeldin. Representative flow cytometry analysis out of 5. (**g**) Histograms summarizing percentages of CD45.1 cells expressing a given cytokine. n = 10 mice for CpG-pre-pDC recipients, n = 3 mice for PBS-pre-pDC recipients, mean ± s.e.m., data are from two cumulated experiments. *, *p* = 0.0111 for GM-CSF, *, *p* = 0.0176, for IL-27, by unpaired Students’*t*-test (**h**) CpG-pre-pDCs were isolated from the BM culture of either WT or IL-27^−/−^ mice in presence of 1 µg/ml CpG-B and 80,000 of them injected i.v into EAE immunized mice at d-12. Clinical score (mean ± s.e.m.) was assessed until d-45. n = 7 for the IL-27^−/−^ CpG-pre-pDCs treated group, n = 8 for the WT CpG-pre-pDCs treated group, n = 14 for the control group, injected with PBS. Statistical analysis was performed using two-way ANOVA with Bonferroni post-test. Controls vs + IL-27^−/−^ CpG-pre-pDCs, d12–21, **p = 0.0024, NS afterwards, Controls vs + WT CpG-pre-pDCs, d15–d35, **, p = 0.002, d40–d44, p = 0.0012.

CpG-pre-pDCs and PBS-prepDCs prepared from congenic CD45.1 or actin-GFP^+^ donor mice were used to track their migration and differentiation profile in CD45.2 C57Bl/6J recipients in which they were adoptively transferred at day-12 after immunization. Six days after their adoptive transfer, the vast majority of CD45.1 cells in CpG-pre-pDC recipients were found in the spinal cord, while only very few were found in the spleen and the cervical lymph nodes of the recipient mice (Fig. 3a). PBS-pre-pDCs exhibited similar specificity of migration to the spinal cord but migrated half less than their CpG-activated counterparts (Fig. 3a). In the spinal cord, FACS analysis of gated GFP^+^ cells showed that both migrated CpG- and PBS-pre-pDCs exhibited a c-kit^+^Sca-1^+^B220^low^Siglec-H^+^CD11c^low^ PDCA-1^+^ phenotype similar to their pre-injection phenotype (Fig. 3b). In addition to their conserved immature phenotype, we further assessed whether protective CpG-pre-pDCs retained an immature functional profile. In keeping with their low IFN-α response to CpG stimulation, CpG-pre-pDCs expressed low levels of IRF7, the master positive regulator of IFN-α response to TLR-9 stimulation, compared to mature spleen pDCs (Fig. 3c). Moreover, CD45.1 adoptively transferred CpG-pre-pDCs retained their significantly reduced IRF7 expression after migrating to the spinal cord in CD45.2 recipients (Fig. 3d,e), as compared to host CD45.2 PDCA-1^+^ cells in the same tissue in CD45.2 recipients and to CD45.2 PDCA-1^+^ cells in control mice with EAE injected with PBS medium only. MHC class II and co-stimulatory molecules CD80 and CD86 expression did not change at the surface of CpG-pre-pDCs upon migration but CD40 expression was nearly lost (Supplementary Fig. S2). Thus, CpG activation confers improved selective migration to the inflamed spinal cord to pDC precursors where they retain their precursor stage of differentiation and remain limited in their IFN-α response capacity.

### Role of IL-27 production by CpG-pre-pDCs in the protection against EAE

CpG- and PBS-pre-pDCs derived from CD45.1 or GFP donor mice were used to assess their cytokine production profile after migration. Notably, CD45.1/GFP^+^ CpG-pre-pDCs recovered from the spinal cord of recipients at day-18 demonstrated in response to PMA + ionomycin stimulation intracytoplasmic expressions of GM-CSF and of the anti-inflammatory cytokine IL-27 in recipients that were enhanced relative to PBS-pre-pDCs, but no more displayed TGF-β expression (Fig. 3f,g).

To investigate the functional role of the anti-inflammatory cytokine IL-27 characterizing the protective progenitors migrated into the spinal cord, CpG-pre-pDCs were prepared from IL-27p28 genetically deficient donor mice. Recipients of IL-27^−/−^ progenitors were protected against EAE similar to recipients of WT CpG-pre-pDCs until day-22, but disease gradually worsened thereafter to reach the clinical score of controls (Fig. 3h). IL-27 thus plays a late role in the protection against EAE afforded by CpG-pre-pDCs. These data may further suggest that adoptively transferred pDC progenitors persist at least until approximately d-22 and probably longer in recipients.

### Modulation of host cells in the spinal cord of pDC precursors recipients

**Figure 4:**
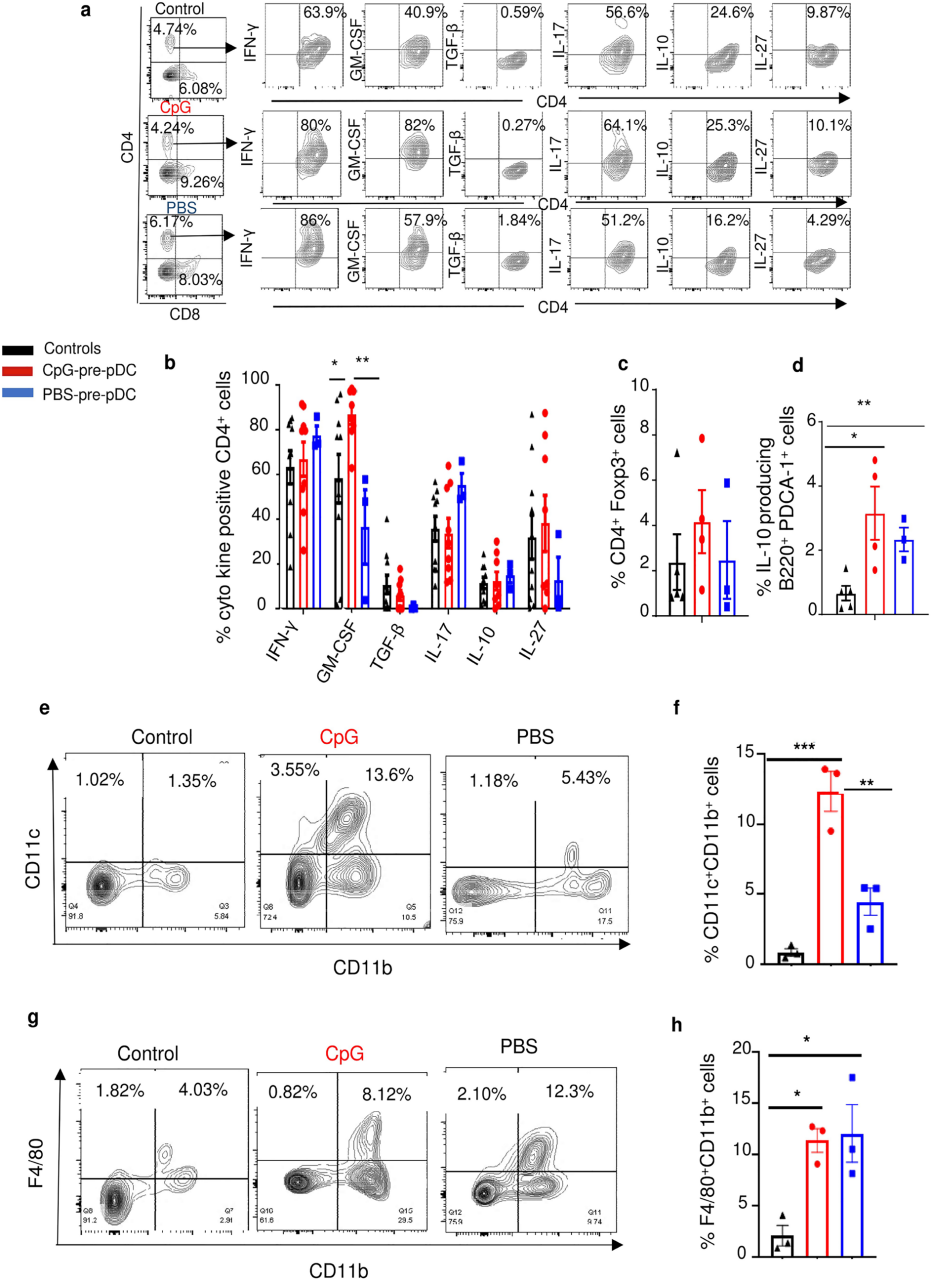
Analysis of host cells in the spinal cord. Cells recovered from the spinal cord of mice immunized for EAE, either controls injected with PBS medium but no progenitors or injected with CpG- or PBS-pre-pDCs were analyzed at d-18 for their intracytoplasmic expression of cytokines after 4 h activation with PMA/ionomycin in presence of brefeldin using isotype controls. (**a**) Representative FACS profiles of CD4^+^ T-cells. (**b**) Histogram summary of percentages of cytokine-expressing CD4^+^ T-cells in controls (n = 9) and in CpG-pre-pDC (n = 9) and PBS-pre-pDC (n = 3) recipients. Data from two cumulated experiments are expressed as mean ± s.e.m.. Statistical analysis was performed using unpaired Student’s t-test: GM-CSF: Controls vs + CpG-pre-pDCs, *, *p* = 0.0395, + PBS-pre-pDCs vs + CpG-pre-pDCs, **, *p* = 0.0020. Percentages of (**c**) Tregs (CD4^+^ Foxp3^+^ cells) (NS) and of (**d**) IL-10^+^ B cells (B220^+^PDCA-1^−^ cells) were analyzed in the spinal cord of control mice with EAE injected with PBS (n = 5), and in CpG-pre-pDC (n = 5) and PBS-pre-pDC (n = 3) recipients. Mean ± s.e.m. Statistical analysis using one-way ANOVA with Tukey’s post-test indicated significant differences with *, *p* = 0.0166, **, *p* = 0.0034. (**e**–**h**) Myeloid cells were analyzed. Results shown are representative FACS profiles (**e**) and summary histograms (mean ± s.e.m.) of percentages of CD11c^+^CD11b^+^ cells (**f**) and F4/80^+^CD11b^+^ macrophages (**g**, **h**), (**e**–**h**) *, *p* = 0.028,**, *p* = 0.0035,***, *p* = 0.0005, analyzed by one-way ANOVA with Tukey’s post-test.

We studied the impact of the adoptive transfer of CpG-versus PBS-pre-pDCs on host cells in the spinal cord of recipients, at d-18, compared to controls with EAE injected with PBS medium but no progenitors. Surprisingly, CpG-pre-pDC recipients only showed enhanced percentages of GM-CSF expressing CD4^+^ T-cells relative to CD4^+^ T-cells in other groups while no significant changes were observed in the proportion of CD4^+^ T-cells expressing IFN-γ, IL-17, IL-10, IL-27 or TGF-β (Fig. 4a,b) nor in the percentages of Foxp3^+^ CD4^+^ T-cells (Fig. 4c). Instead, in keeping with reports showing that GM-CSF, here produced by both migrated CpG-pre-pDCs and CD4^+^ T-cells in their recipients, may exert regulatory effects by triggering accumulation of IL-10 producing B-cells (B10)^33,34^ and of CD11c^+^CD11b^+^ tolerogenic DCs^35,36^, we found that these populations were significantly accumulated in the spinal cord of CpG-pre-pDC recipients compared to controls for the B10 (Fig. 4d) and to both controls and PBS-pre-pDC recipients for the DCs (Fig. 4e,f). Finally, F4/80^+^CD11b^+^ macrophages were similarly enhanced compared to controls in recipients of both PBS- and CpG-pre-pDCs (Fig. 4g,h).

### Adoptive transfer of CpG-pre-pDCs transiently modifies host pDCs

**Figure 5:**
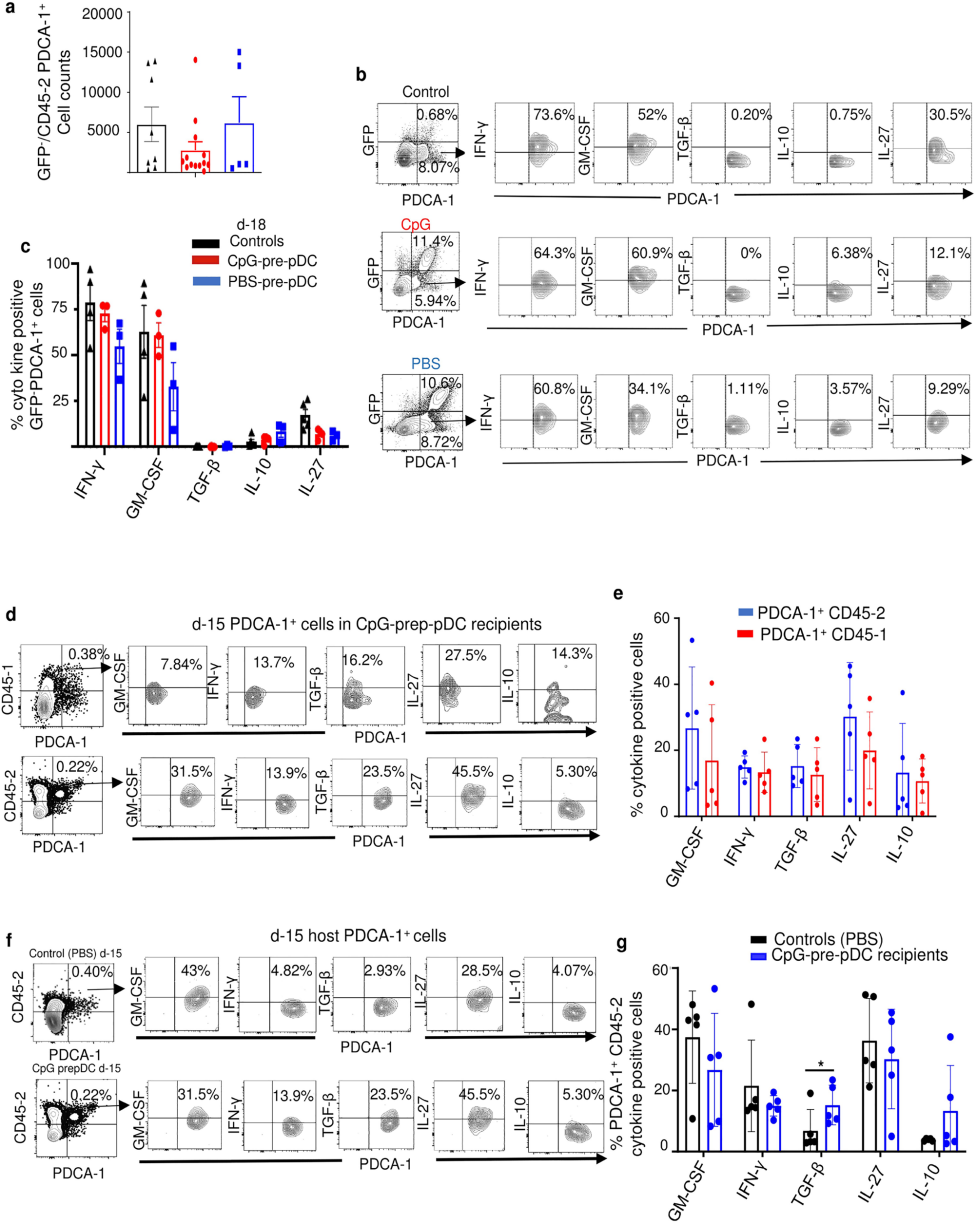
Quantitative and qualitative assessment of PDCA-1^+^ cells in the spinal cord. (**a**) Flow cytometry analysis of host GFP^−^/CD45.2 PDCA-1^+^ cell counts at d-18 in the spinal cord of control mice with EAE injected with PBS (n = 8) relative to those in CpG-pre-pDC (n = 13) and PBS-pre-pDC (n = 5) recipients. Data from two cumulated experiments. No significant differences were found using one-way ANOVA with Bonferroni post-test. (**b**, **c**) Cytokine expression measured using isotype controls, after 4 h activation with PMA/ionomycin in presence of brefeldin, by host GFP^−^PDCA-1^+^ cells at d-18 in the spinal cord of the same three groups of mice (n = 4 controls, n = 3 CpG-pre-pDC recipients and n = 3 PBS-pre-pDC recipients). Shown are representative FACS profiles (**b**) and histograms of percentages expressed as mean ± s.e.m. (**c**) No significant statistical differences were found using one-way ANOVA with Bonferroni post-test. (**d**, **e**) Compared cytokine expression analysis at d-15 in CD45.1 progenitor-derived and CD45.2 host-derived PDCA-1^+^ cells of the spinal cord of CpG-pre-pDC recipients. Shown are representative FACS profiles (**d**) and histograms of percentages (**e**) expressed as mean ± s.e.m. (n = 5 mice/group). No significant differences using Students’ *t*-test. (**f**, **g**) Compared cytokine expression analysis at d-15 in host CD45.2 PDCA-1^+^ cells in control mice with EAE injected with PBS and in CpG-pre-pDC recipients. Shown are representative FACS profiles (**f**) and histograms of percentages (**g**) expressed as mean ± s.e.m. (n = 5 mice). Statistical analysis using Students’ *t*-test, *, *p* = 0.0291.

Contrary to mature pDCs bearing the auto-antigen, reported to induce host pDC accumulation in the spinal cord^18^, neither CpG- nor PBS-pre-pDCs induced significant host pDC accumulation at d-18 (Fig. 5a) nor at d-15 or d-25 (Supplementary Fig. S3).

We further investigated if qualitative changes were induced in host pDCs by the adoptive transfer of pDC progenitors. No significant differences in cytokine expression by GFP-PDCA-1^+^ host pDCs were observed at d-18 among the three groups (controls with EAE not injected with progenitors, CpG-pre-pDC recipients and PBS-pre-pDC recipients) and no TGF-β expression was detectable (Fig. 5b,c). However, TGF-β was detectable earlier at d-15 in CpG-pre-pDC recipients, in PDCA-1^+^ cells either derived from the adoptively transferred CD45.1/GFP^+^ precursors or from the CD45.2 host (Fig. 5d,e), that displayed equivalent overall cytokine expression. Notably, at d-15 of EAE, host CD45.2 PDCA-1^+^ cells expressed more TGF-β when examined in the spinal cord of CpG-pre-pDC recipients than in controls with EAE injected with PBS medium only but no progenitors (Fig. 5f,g). This TGF-β expression both in migrated progenitors and in host pDCs in CpG-pre-pDC recipients is in keeping with the report that TGF-β exposure of pDCs prompts in turn their production of TGF-β^37^. Therefore, adoptively transferred CD45.1 CpG-pre-pDCs retain their TGF-β expression until d-15 and promote TGF-β expression also by host CD45.2 pDCs until that same time.

### Role of TGF-β expressed by CpG-pre-pDCs in the spinal cord in the protection against EAE

**Figure 6:**
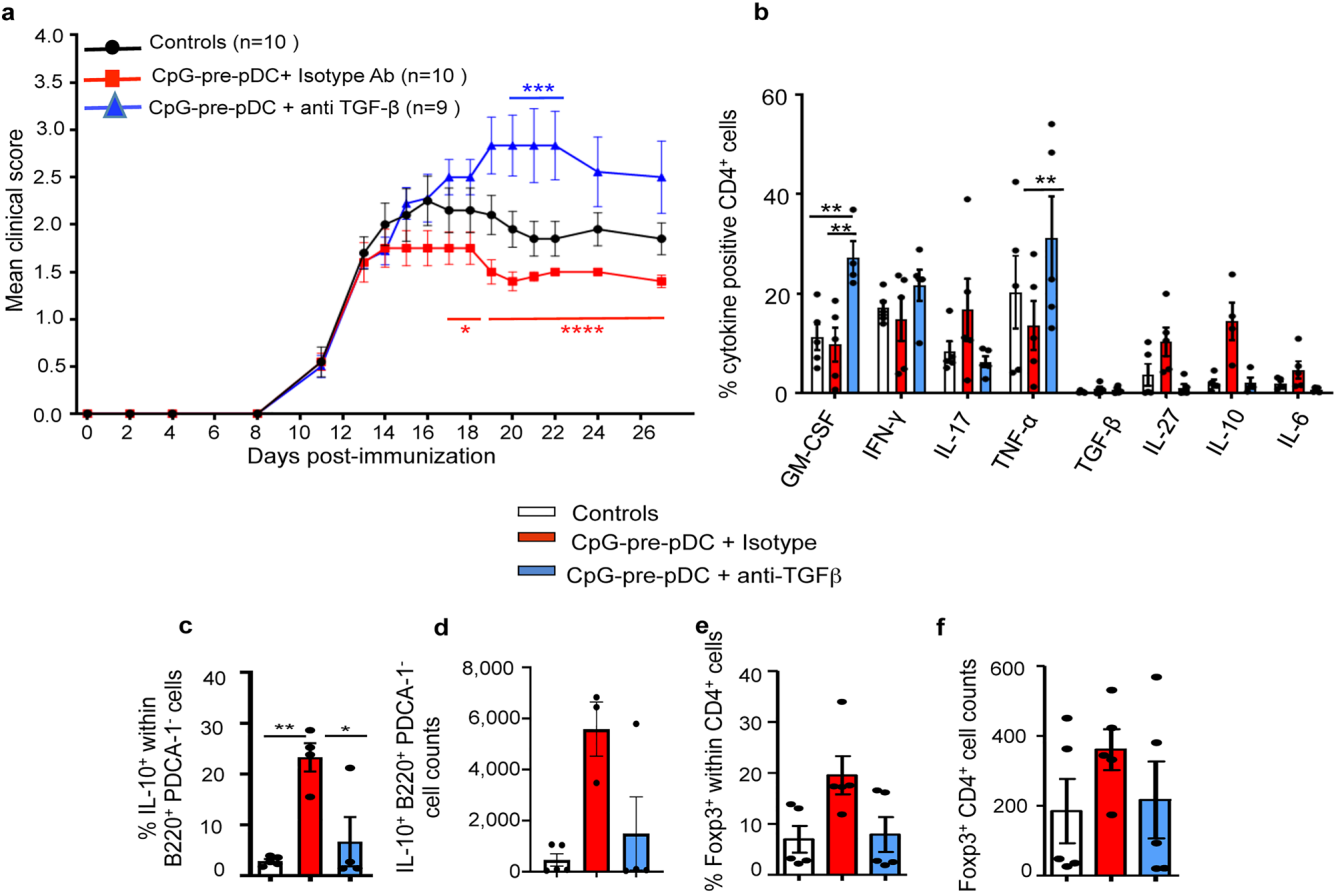
Role of TGF-β in CpG-pre-pDC mediated protection against EAE. (**a**) CpG-pre-pDCs were transfected with a neutralizing anti-TGF-β antibody or control isotype antibody using the Chariot protein transfection vector. 80,000 of resulting cells were injected i.v. to MOG_35-55_ immunized mice at d-12 after immunization. Clinical score (mean ± s.e.m.) was assessed until d-27, n = 10 mice in control and isotype antibody progenitor-treated groups and n = 9 mice in the anti-TGF-β-progenitor-treated group. Statistical analysis was performed using two-way ANOVA with Bonferroni post-test: controls vs CpG-prepDC + isotype, d-19, *p = 0.0352; controls vs CpG-prep-DC + anti-TGF-β, d-19, *, *p* = 0.0144, d20–d22, ***p < 0.0025, d27 *, *p* = 0.0352; CpG-pre-pDCs + isotype vs CpG-pre-pDCs + anti-TGF-β, d17–18, *, *p* = 0.0119, d19–d27, ****, *p* < 0.0001. (**b**) CD4^+^ T-cells isolated at d-27 from the spinal cord of controls or recipients of isotype or neutralizing Ab-transfected CpG-pre-pDCs were analyzed by FACS using FMO controls for their cytokine production after 4 h activation with PMA/ionomycin in presence of brefeldin. Mean ± s.e.m. of percentages of cells expressing a given cytokine, n = 5 mice per group. Statistical analysis was performed using two-way ANOVA with Bonferroni post-tests, for GM-CSF: Controls vs CpG-pre-pDCs + isotype Ab, NS, Controls vs CpG-pre-pDCs + anti-TGF-β, **, *p* = 0.0032, CpG-pre-pDCs + isotype vs + anti-TGF-β, **, *p* = 0.008, *; for TNF-α, CpG-pre-pDCs + isotype vs + anti-TGF-β, **, *p* = 0.0015. (**c**, **d**) Frequency (**c**) and counts (**d**) of B220^+^ PDCA-1^−^ B-cells demonstrating IL-10 production capacity (mean ± s.e.m., n = 5 mice per group, * *p* = 0.01, **, *p* = 0.0015, by one-way ANOVA with Bonferroni post-tests). (**e**, **f**) CD4^+^Foxp3^+^ Tregs frequency (**e**) and cell counts (**f**), (mean ± s.e.m., n = 5 mice per group), N.S. by analysis using Kruskal–Wallis with Dunn’s post-tests.

To evaluate the role of TGF-β expressed selectively by CpG-pre-pDCs, before injection and until d-15, in their protection against EAE, we sought to deprive TGF-β from the adoptively transferred CpG-pre-pDCs. Given that TGF-β deficient mice are not viable, and technics based on the use of RNAs are susceptible to activate pDCs, we inserted a neutralizing antibody directed against TGF-β into the progenitors with the help of a protein transfection vector Chariot. We previously reported efficient neutralization of a chemokine released by adoptively transferred DCs^38^ using the same experimental method. We now confirmed using a bioassay for TGF-β^39^ based on inhibition of IL-4 induced HT-2 cell proliferation (Supplementary Fig. S4), that while TGF-β bioactivity was detectable in the lysate of isotype-transfected CpG-pre-pDCs, insertion within CpG-pre-pDCs of the vector coupled with a TGF-β neutralizing antibody reduced by approximately 40% the TGF-β bioactivity in the precursor lysate, thereby suggesting at least partial neutralization of TGF-β by the Chariot-antibody complex within pDC precursors. In addition, the vector-antibody complex insertion procedure did not affect the viability of CpG-pre-pDCs, nor altered their phenotype or their cytokine production profile (Supplementary Fig. S5), except for two-fold reduced TGF-β detectable levels, suggesting that half of the TGF-β content was complexed by the Chariot-coupled antibody. Notably, while CpG-pre-pDCs in which an isotype antibody had been inserted were still able to provide some protection against EAE, CpG-pre-pDCs containing a TGF-β-neutralizing antibody lost their protective capacity and even worsened the disease score (Fig. 6a). Analysis at day-25 showed that such aggravation of disease correlated with a higher frequency of CD4^+^ T-cells from the spinal cord producing GM-CSF and TNF-α, relative to recipients of CpG-pre-pDCs bearing the isotype antibody (Fig. 6b). In addition, enhanced percentages, but not numbers, of B10 cells in the spinal cord of recipients of CpG-pre-DCs in which an isotype Ab was inserted was lost when an anti-TGF-β antibody was inserted instead (Fig. 6c,d). However, the tendency to enhanced percentages or numbers of Foxp3 Tregs (Fig. 6e,f) did not reach statistical significance among groups.

### CpG-pre-pDCs protect against all phases of EAE

**Figure 7:**
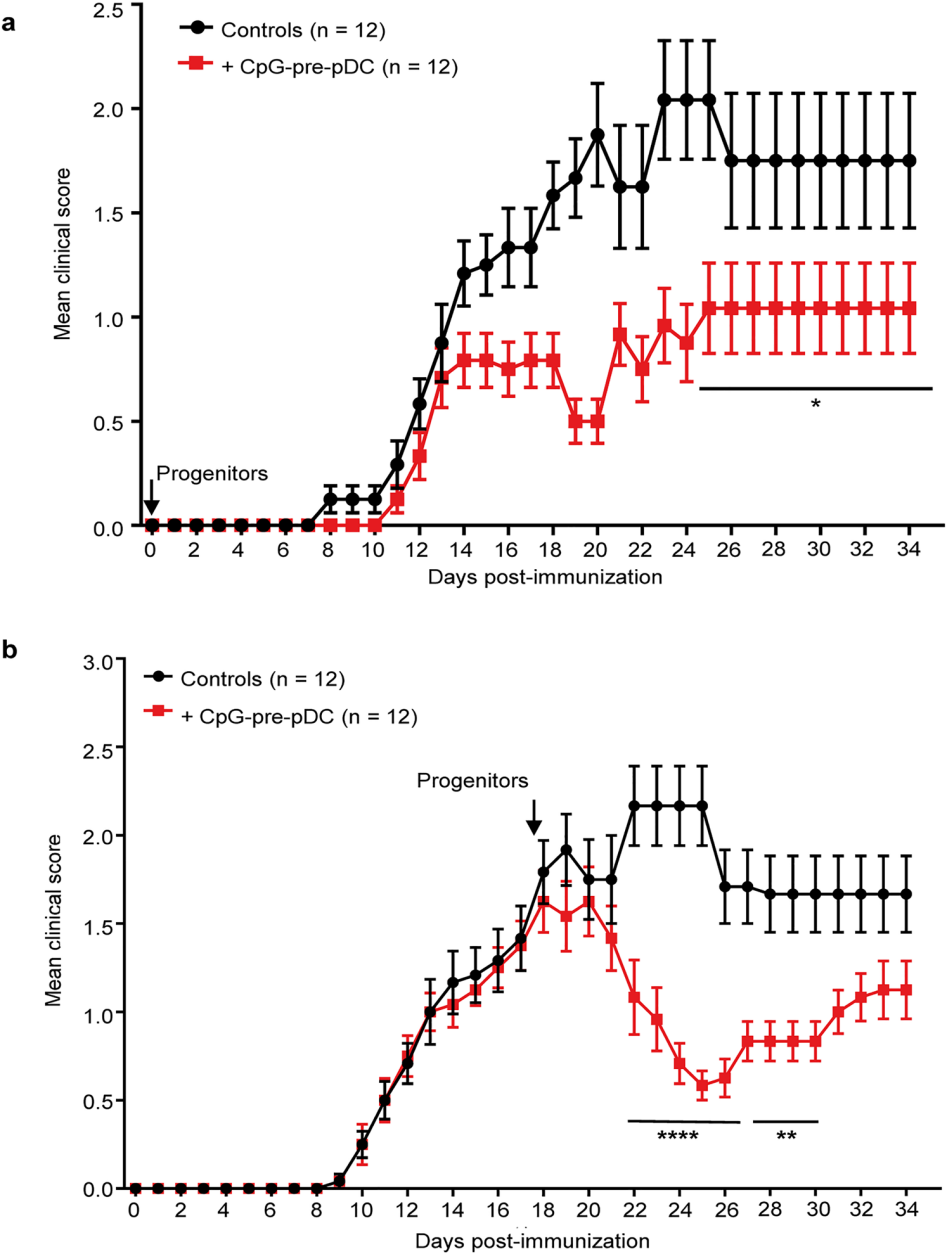
Extended therapeutic window of CpG-pre-pDCs against EAE. CpG-pre-pDCs, 80,000 per recipient, were adoptively transferred either (**a**) at d-0 (n = 12 mice per group), or (**b**) at d-17 (n = 12 mice per group) after immunization. Clinical scores were assessed until day-35. Data are from 2 cumulated experiments. Statistical analysis was performed using two-way ANOVA with Bonferroni post-test, (**a**) d25–d34, *, *p* = 0.0453, (**b**) d22–d26, ****, *p* < 0.0001, d27–d30, **, *p* = 0.0021.

Finally, we further explored the therapeutic window of CpG-pre-pDCs in the course of EAE. When 80,000 CpG-pre-pDCs were adoptively transferred at the time of immunization, at day-0, recipients remained protected until the end of the experiment, i.e. day-35 (Fig. 7a). More importantly, when 80,000 CpG-pre-pDCs were transferred at the peak of the disease, i.e. day-17, the clinical scores were stabilized within the 3 following days and thereafter decreased by approximately 60% from day-22 until day-32 (Fig. 7b). Therefore, the CpG-activated pDC precursors demonstrate a unique therapeutic potential against EAE at any stage of disease.

**Figure 8:**
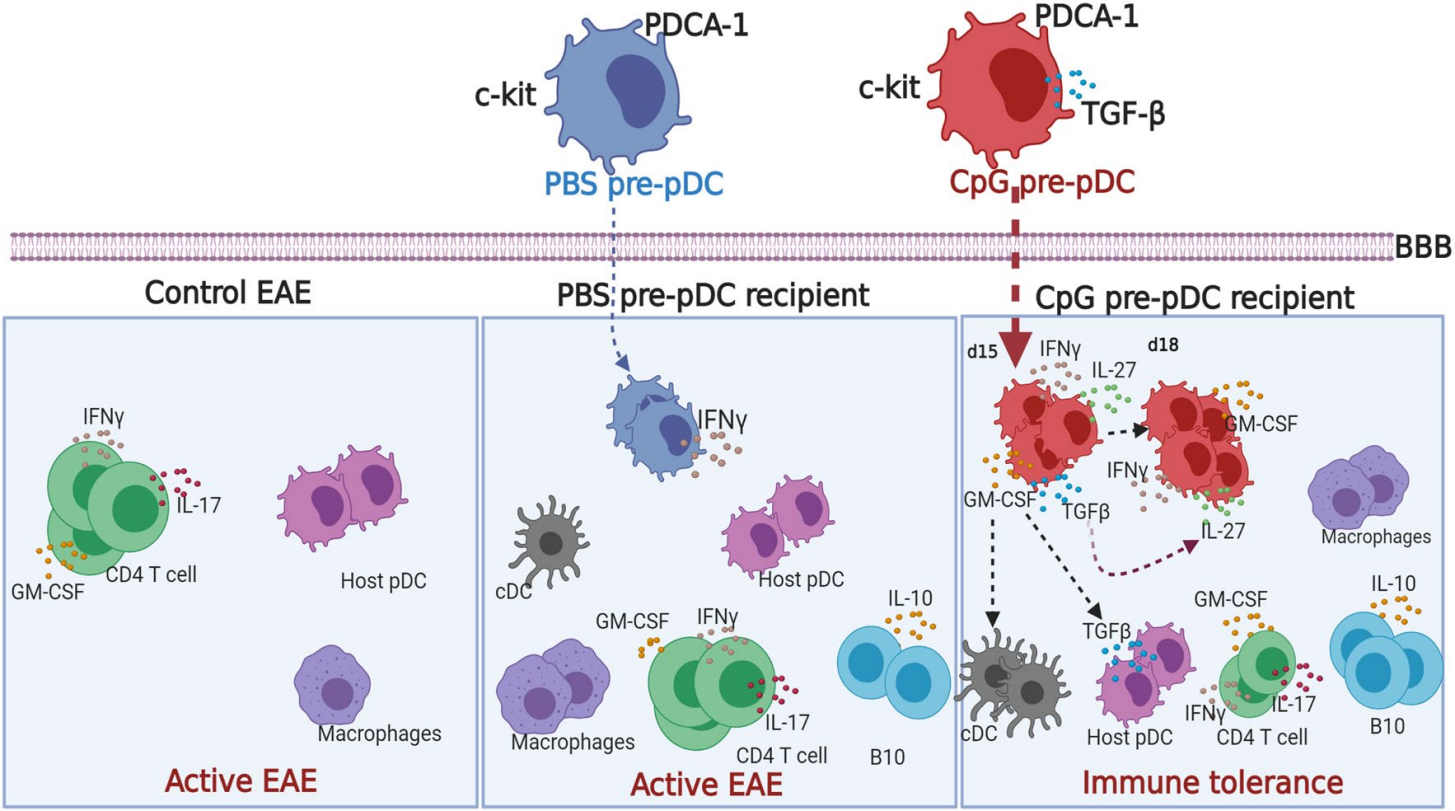
Graphical representation of cellular and molecular mechanisms of the protective effect of CpG-pre-pDCs against EAE. Upon adoptive transfer of pDC progenitors at d-12, the onset of clinical signs, TGF-β released only by CpG-pre-pDCs in the first 3 days in the spinal cord (light blue zone) of mice protects against EAE by prompting host pDCs to release in turn TGF-β and promoting accumulation of CD11c^+^CD11b^+^ cDCs and IL-10^+^ B cells. CpG-pre-pDCs, once migrated to the spinal cord, additionally release IL-27 that from day-22 onwards, ensures late protection against EAE. *BBB* blood brain barrier. The figure was created using Biorender.com.

A graphical summary of cellular and molecular mechanisms accounting for the protective effect of CpG-pre-pDCs against EAE is displayed in Fig. 8.

## Discussion

CpG-activated pDC precursors represent a powerful regulatory cell population that provides protection against EAE disease at remarkably low cell counts, compared to most mature cell types so far tested. Contrary to Tregs that tend to lose their suppressive properties in an inflammatory environment^11^, CpG-pre-pDCs are equally protective at all disease stages, i.e. initiation, onset of clinical signs and at peak of disease, therefore displaying a promising therapeutic potential, highly desirable for a robust cell therapy.

The susceptibility of CpG-pre-pDCs to adopt regulatory functions selectively upon either in vivo or in vitro stimulation with the TLR-9 agonist CpG-B differs from the report that CpG-B was instead unable to confer protective properties against EAE onto mature pDCs^18^ where loading with antigen was strictly required to acquire therapeutic properties. Six days after injection, CpG-pre-pDCs have essentially migrated to the inflamed spinal cord, a feature they share with reported myelin antigen-loaded pDCs^18^ and do so approximately twice more than PBS-pre-pDCs. However, it is noteworthy that CpG-pre-pDCs, even after migration to the inflamed spinal cord, kept both an immature phenotype with c-kit expression as well as an immature functional profile retaining low expression levels of IRF7, a master positive regulator of IFN-α response to TLR-9 stimulation^40,41^. Therefore, the immature state of pDC progenitors is a key characteristic of their tolerogenic properties that clearly differ from those of mature pDCs.

Nevertheless, the functionally immature state of pDCs is not sufficient to confer a pro-tolerance profile as injections of PBS-pre-pDCs (our data) as well as of immature pDCs^42^ in vivo were unable to induce antigen-specific tolerance. Notably, CpG enhanced the pDC precursor migration to the spinal cord, in correlation with enhanced CD29 expression, enabling CpG-pre-pDCs to exert their tolerogenic role in the CNS where neuroinflammation takes place^25^. Additionally, CpG activation induced their TGF-β expression that appeared crucial for their regulatory properties. Altogether, our data demonstrate that both the precursor stage of CpG-pre-pDCs and activation by CpG are crucial for developing a tolerogenic cell population within the inflamed spinal cord.

Once injected, CpG-pre-pDCs produce both TGF- β and IL-27. We demonstrated that each one of these regulatory cytokines plays a functional role, with TGF-β being expressed and required from the day of their adoptive transfer whereas IL-27, a member of the IL-12 family^43^, is induced after migration and appears to play a non-redundant key role in the late events taking place 10 days after the transfer. Higher expression of both IL-27 and its receptor have been found at the effector phases of EAE, disease peak, and relapse^44^. This sequential action of TGF-β and IL-27 is in line with the capacity of TGF-β to induce IL-27 in pDCs^45^.

The tolerogenic role of TGF-β produced by pDCs has been reported in various immune settings, including tumor defense as well as autoimmune diseases. TGF-β production, induced by GM-CSF and TNF-α in tumors, is a recognized feature of intra-tumoral pDCs that promote tumor escape^37^. In T1D, viral infection triggered the production of TGF-β by pancreatic pDCs via activation of NKT cells^46^ involving both IL-10 and PDL-1, resulting in lower T1D incidence. Moreover, TGF-β controls IRF7 in TLR-9 activation of pDCs^47^ and we observed reduced IRF7 expression in CpG-pre-pDCs at their emergence and after their migration into the spinal cord. Furthermore, contrasting with the endogenous pDC recruitment observed by Duraes et al.^18^ that was essential in the protective effect of myelin-Ag exposed pDCs, TGF-β expressing CpG-pre-pDCs did not recruit endogenous pDCs into the spinal cord but instead prompted local host pDCs to transiently produce TGF-β.

Although both TGF-β and IL-27 have the capacity to act directly on effector T-cells, there was no major modulation of cytokine production by T cells in the spinal cord of protected CpG-pre-pDC recipients, except for a transiently enhanced GM-CSF production, apparent at d-18 but no more at d-25. While GM-CSF is implicated in neuropathogenesis in EAE^48,49^, it was also reported to exert immunoregulatory effects by triggering accumulation of tolerogenic CD11c^+^CD11b^+^ DCs^36^ and of regulatory B10 cells^33,34^. In addition, it was previously reported that pDCs induced IL-10 producing Bregs (B10)^50^. Interestingly, both host B10 and DCs likewise accumulated in the spinal cord of CpG-pre-pDC recipients, but not in PBS-pre-pDC recipients, compared to controls with EAE injected with PBS medium but no pDC precursors. Therefore, various host cells may contribute to the long-lasting protection provided by CpG-pre-pDCs.

In addition to TGF-β and IL-27 that play a major role in the protection against EAE afforded by CpG-pre-pDCs, the progenitors also express GM-CSF that despite very low levels when measured by ELISA in their supernatants, was clearly detectable by intracellular flow cytometry (Supplementary Fig. S5). Upon migration into the inflamed spinal cord, CpG-pre-pDCs express significantly more GM-CSF as well as IL-27 than PBS-pre-pDCs (Fig. 3f,g). GM-CSF expression by pDCs has been reported^51^ to be induced by CpG but its role remains to be elucidated. Finally, CpG also induced PDL-1 expression on CpG-pre-pDCs (Supplementary Fig. S6). The role of this surface molecule warrants further investigation.

The cellular and molecular mechanisms of protection exerted by CpG-pre-pDCs against EAE are summarized in Fig. 8, highlighting the sequential role of TGF-β and IL-27 as well as the accumulation of host regulatory cells such as B10, cDCs and TGF-β producing host pDCs.

Altogether, data herein suggest that early CpG activation of pDC precursors within the BM generates unique powerful regulatory cells that are effective at very low cell counts at all phases of EAE and migrate selectively to the site of the autoimmune response. Compared with previous demonstrations regarding mainly progenitors in the monocyte and B-cell lineages^5,9,10^, as well as progenitors still at the multipotent stage of differentiation^6–8^, we demonstrate that innate stimuli prime BM pDC hematopoietic progenitors into a tolerogenic function which remains imprinted in inflammatory settings. Whether equivalent therapeutic human pDC precursors exist and display protective properties against MS, either on their own or in the context of autologous HSCT, already implemented in patients with severe MS^52–56^, might be worth investigating.

## Methods

### Mice

WT CD45.2 C57Bl/6J mice were obtained from Janvier Laboratories (Le Genest Saint Isle, France) and housed in our animal facility for at least 2 weeks before EAE induction. Congenic CD45.1 and GFP-actin KI C57Bl/6J mice, all backcrossed for at least ten generations, were bred in our accredited animal facility at Institut Necker Enfants Malades under pathogen-free conditions. IL-27p28^−/−^ C57Bl/6J mice were raised at the Medical College of Wisconsin department of Medicine. Female 10-week-old C57BL/6J mice were used as a model for MOG_35–55_-induced EAE and received intravenous progenitor cell transfers at various times after immunization. The study was carried out in compliance with the ARRIVE guidelines.

### EAE induction

Active EAE was induced in 10-week-old female mice by s.c. immunization at two sites, upper and lower back, with 200 µg MOG_35–55_ peptide emulsified in CFA containing 400 µg heat-killed Mycobacteria tuberculosis H37Ra (Hooke Laboratories, Lawrence, MA) on day 0. In addition, mice received 300 ng pertussis toxin (Hooke Laboratories) i.p. in 0.1 ml per mouse on days 0 and 1. Clinical signs of EAE were assessed daily on a 0–5 scale defined as follows: 0, no obvious changes in motor function compared to non-immunized mice; 0.5, tip of tail is limp; 1, limp tail; 1.5, limp tail and unstable gait; 2, limp tail and hind leg inhibition; 2.5, limp tail and dragging of hind legs; 3, limp tail and complete paralysis of hind legs or paralysis of one front and one hind leg; 3.5, limp tail and complete paralysis of hind legs that are together on one side of body; 4, limp tail, complete hind leg and partial front leg paralysis; mouse is still minimally moving and appears feeding; 4.5, complete hind leg and partial front leg paralysis; no movement around the cage, mouse is not alert; 5, severe paralysis or death. Mice with score ≥ 4 for 2 consecutive days and mice with score 5 were euthanized and a score of 5 retained for the rest of the experiment.

### Sorting of TLR-activated bone marrow progenitors

Bone marrow (BM) cells removed from tibiae, femurs and hips of 8- to 14-week-old C57BL/6J mice were incubated in low endotoxin RPMI-1640 medium (Fisher Scientific, Illkirch, France) supplemented with 10% (vol/vol) FCS and 1% antibiotics (penicillin and streptomycin) for 18 h with 1 μg/ml of the oligodeoxynucleotide CpG 1668 (CpG-B) (Eurogentec, Angers, France) or with the respective agonists of TLR1-9, supplied in a commercial kit (InvivoGen, Toulouse, France), including: Pam3CSK4 (0.5 μg/ml), FSLI (1 μg/ml), HKLM (2 × 10^6^ cells/ml), Poly I:C (HMW) (5 μg/ml), Poly I:C (LMW) (5 μg/ml), LPS-EK standard (1 μg/ml), FLA-ST standard (1 μg/ml), ssRNA40/LyoVec (2 μg/ml) as well as CpG 1826 (CpG-B) (1 μg/ml). c-kit^+^ BM cells were sorted by immuno-magnetic separation using a RoboSep automaton (StemCell Technologies, Grenoble, France). Sorted cells were further stained with appropriate fluorochrome-conjugated mAbs against Sca-1, B220 (BD Biosciences, Le Pont de Claix, France), PDCA-1 (eBioscience, Paris, France or BioLegend, London, UK) and electronically sorted as a c-kit^+^Sca-1^+^B220^+^PDCA-1^+^ cell subset using a FACS-Aria IIIu (BD Biosciences).

### Isolation of immune cells from the spinal cord

Spinal cords isolated from control and CpG- or PBS-pre-pDC-recipient mice were passed through a 70 μm cell strainer, using the back side of a syringe plunger to smash the tissue. Cells were then resuspended in 3–5 ml of 30% Percoll underlayed with the same volume of 70% Percoll (in PBS) and centrifuged for 20 min at 1300*g* without brakes to form a smooth interface. Cells were collected with a Pasteur pipette and diluted ten times with complete RPMI 10% FCS medium. After centrifugation, cells were resuspended in 2–3 ml of complete medium and further stained with appropriately labeled mAbs.

### Staining of cells for flow cytometry analysis

To block nonspecific Fc receptor binding, cells were pre-incubated for 10 min at room temperature with FcR blocker 2.4G2 mAb. Cells were then stained with appropriately labeled mAbs against CD4, B220, MHC II, PDCA-1, CD115, Siglec-H, Ly6D, PDL-1, PDL-2 (eBioscience, ThermoFisher Scientific, Montigny-le-Bretonneux, France), c-Kit (CD117) (BioLegend, San Diego, CA), CD127, CD11c, Sca-1 (anti-Ly6A/E), CD40, CD80, CD86, CD45.1, CD45.2 (BD Bioscience/Pharmingen, Le Pont-de-Claix, France), CD2, CD81 (Sony, Weybridge, Surrey, UK) or GFP (ThermoFisher Scientific). Nuclear Foxp3, Id2, IRF7, IRF8 expression was measured by FACS analysis as per the manufacturer’s instructions (eBioscience, ThermoFisher Scientific). Positive cells were defined using a “fluorescence minus one” (FMO) sample or an isotype control as indicated in figure legends. Nuclear factor expression of E2-2 and PU-1 was measured by Quantigene FlowRNA (Affymetrix, ThermoFisher Scientific) as per the manufacturer’s instructions.

### Cytokine assays

Intra-cytoplasmic expression of cytokines by cells, either just after cell-sorting or recovered from the spinal cord was assessed after a 4-h stimulation with PMA (10 ng/ml) plus ionomycin (500 ng/ml) in the presence of Brefeldin A (2 mg/ml), followed by fixation/permeabilization with PFA/saponin and subsequent staining with specific antibodies including PE-labeled anti-TGF-β, PE-labeled anti-IL-27p28, PE-labeled anti-GM-CSF, APC-labeled anti-IL-10, APC-labeled anti–IFN-γ (eBioscience), APC-labeled anti-IL-17 (BD bioscience), FITC-labeled anti-IL-6 and FITC-labeled anti-TNF-α (Biolegend). Positive cells were defined using isotype Ab stained controls (from BD Biosciences and eBioscience) or FMO sample, as indicated in figure legends. Membrane and intracellular antigen expression was analyzed in a FACS Canto II cytometer (BD Biosciences) using FlowJo software (Treestar, Ashland, OR).

Cytokine production by PBS- and CpG-pre-pDCs was measured in supernatant after 4 h-activation of the cell-sorted precursors with PMA + ionomycin by multiplex ELISA using LegendPlex detection reagents from BioLegend. IFN-α production was measured by LegendPlex ELISA (BioLegend) after 24 h stimulation of PBS- and CpG-pre-pDCs with R848 (0.5 μg/ml), or 1 μg/ml CpG-B (Eurogentec, Angers, France), CpG-P (Miltenyi Biotec, Paris, France), LPS (InVivogen) and PBS.

### TGF-β neutralization with Chariot vector

0.1 mg Chariot (Active Motif, La Hupe, Belgium) was combined with 50 µg of either LEAF purified anti-TGF-β neutralizing antibody (clone 2D11.16.8) (BioXCell, West Lebanon, NH) or IgG1 isotype control (Biolegend) according to the manufacturer’s instructions. Electronically sorted CpG-pre-pDCs were incubated with the Chariot construct for 20 min and thoroughly rinsed in PBS alone. Transfected cells were then injected intravenously in PBS, 2% FCS.

### Study approval

All experiments were conducted according to the EU Directive 2010/63/EU for animal experiments under an animal study proposal approved by the Paris Descartes University Ethical Committee for Animal Experimentation and the French Ministry of Research and Higher Education, number 3846-2015070622031545v4.

### Statistics

Statistical analysis was performed using GraphPad Prism (GraphPad Software, La Jolla, CA). Normality and variance equality were assessed for every data set with D’Agostino-Pearson and F Test respectively. Disease curves and multiple cytokine production were analyzed using two-way ANOVA test, with Bonferroni post-test and Geisser-Greenhouse correction when needed. Cell proportions were analyzed using one way ANOVA with Bonferroni post-test. Cytokine production were analyzed by Kruskal–Wallis with Dunn’s post-test. MFI comparisons were analyzed with unpaired *t*-test. Data are shown as mean ± s.e.m.. *P* < 0.05 was considered statistically significant.

## Supplementary Information

Supplementary Information.

## Data Availability

All data are available upon reasonable request.

## References

[1] Massberg S (2007). Immunosurveillance by hematopoietic progenitor cells trafficking through blood, lymph, and peripheral tissues. Cell.

[2] Kaufmann E (2018). BCG educates hematopoietic stem cells to generate protective innate immunity against tuberculosis. Cell.

[3] Naik S, Larsen SB, Cowley CJ, Fuchs E (2018). Two to tango: Dialog between and immunity and stem cells in health disease. Cell.

[4] Mitroulis I (2018). Modulation of myelopoiesis progenitors is an integral component of trained immunity. Cell.

[5] Askenase MH (2015). Bone-marrow-resident NK cells prime monocytes for regulatory function during infection. Immunity.

[6] Kared H (2006). Jagged2-expressing hematopoietic progenitors promote regulatory T cell expansion in the periphery through notch signaling. Immunity.

[7] Kared H (2008). Role of GM-CSF in tolerance induction by mobilized hematopoietic progenitors. Blood.

[8] Korniotis, S. *et al.* Mobilized multipotent hematopoietic progenitors stabilize and expand regulatory T-cells to protect against autoimmune encephalomyelitis. *Front. Immunol.* **11**, 607175 (2020). 10.3389/fimmu.2020.607175 PMC7786289 33424854

[9] Korniotis S (2016). Treatment of ongoing autoimmune encephalomyelitis with activated B-cell progenitors maturing into regulatory B cells. Nat. Commun..

[10] Montandon R (2013). Innate pro-B-cell progenitors protect against type 1 diabetes by regulating autoimmune effector T cells. Proc. Natl. Acad. Sci. USA.

[11] Bailey-Bucktrout SL (2013). Self-antigen-driven activation induces instability of regulatory T cells during an inflammatory autoimmune response. Immunity.

[12] Pashenkov M (2001). Two subsets of dendritic cells are present in human cerebrospinal fluid. Brain.

[13] Stasiolek M (2006). Impaired maturation and altered regulatory function of plasmacytoid dendritic cells in multiple sclerosis. Brain.

[14] Bailey-Bucktrout SL (2008). Cutting edge: Central nervous system plasmacytoid dendritic cells regulate the severity of relapsing experimental autoimmune encephalomyelitis. J. Immunol..

[15] Ioannou M, Alissafi T, Boon L, Boumpas D, Verginis P (2013). In vivo ablation of plasmacytoid dendritic cells inhibits autoimmunity through expansion of myeloid-derived suppressor cells. J. Immunol..

[16] Isaksson M (2009). Plasmacytoid DC promote priming of autoimmune Th17 cells and EAE. Eur. J. Immunol..

[17] Irla M (2010). MHC class II-restricted antigen presentation by plasmacytoid dendritic cells inhibits T cell-mediated autoimmunity. J. Exp. Med..

[18] Duraes FV (2016). pDC therapy induces recovery from EAE by recruiting endogenous pDC to sites of CNS inflammation. J. Autoimmun..

[19] Manz MG (2018). Plasmacytoid dendritic cells: Origin matters. Nat. Immunol..

[20] Rodrigues PF (2018). Distinct progenitor lineages contribute to the heterogeneity of plasmacytoid dendritic cells. Nat. Immunol..

[21] Dress RJ (2019). Plasmacytoid dendritic cells develop from Ly6D+ lymphoid progenitors distinct from the myeloid lineage. Nat. Immunol..

[22] Prinz M, Priller J (2010). Tickets to the brain: Role of CCR2 and CX3CR1 in myeloid cell entry in the CNS. J. Neuroimmunol..

[23] Kohler RE (2008). Antagonism of the chemokine receptors CXCR3 and CXCR4 reduces the pathology of experimental autoimmune encephalomyelitis. Brain Pathol..

[24] McCandless EE, Wang Q, Woerner BM, Harper JM, Klein RS (2006). CXCL12 limits inflammation by localizing mononuclear infiltrates to the perivascular space during experimental autoimmune encephalomyelitis. J. Immunol..

[25] Garnier A (2019). CD49d/CD29-integrin controls the accumulation of plasmacytoid dendritic cells into the CNS during neuroinflammation. Eur. J. Immunol..

[26] Cisse B (2008). Transcription factor E2–2 is an essential and specific regulator of plasmacytoid dendritic cell development. Cell.

[27] Nagasawa M, Schmidlin H, Hazekamp MG, Schotte R, Blom B (2008). Development of human plasmacytoid dendritic cells depends on the combined action of the basic helix-loop-helix factor E2–2 and the Ets factor Spi-*B*. Eur. J. Immunol..

[28] Ghosh HS, Cisse B, Bunin A, Lewis KL, Reizis B (2010). Continuous expression of the transcription factor e2–2 maintains the cell fate of mature plasmacytoid dendritic cells. Immunity.

[29] Reizis B (2019). Plasmacytoid dendritic cells: Development, regulation, and function. Immunity.

[30] Schotte R (2003). The transcription factor Spi-B is expressed in plasmacytoid DC precursors and inhibits T-, B-, and NK-cell development. Blood.

[31] Martín-Martín L (2009). Immunophenotypical, morphologic, and functional characterization of maturation-associated plasmacytoid dendritic cell subsets in normal adult human bone marrow. Transfusion.

[32] Niederquell M (2013). Sca-1 expression defines developmental stages of mouse pDCs that show functional heterogeneity in the endosomal but not lysosomal TLR9 response. Eur. J. Immunol..

[33] Sheng JR, Quan S, Soliven B (2014). CD1d(hi)CD5+ B cells expanded by GM-CSF in vivo suppress experimental autoimmune myasthenia gravis. J. Immunol..

[34] Sheng JR, Quan S, Soliven B (2015). IL-10 derived from CD1dhiCD5^+^ B cells regulates experimental autoimmune myasthenia gravis. J. Neuroimmunol..

[35] Bhattacharya P (2015). Dual role of GM-CSF as a pro-Inflammatory and a regulatory cytokine: Implications for immune therapy. J. Interferon Cytokine Res..

[36] Ganesh BB, Cheatem DM, Sheng JR, Vasu C, Prabhakar BS (2009). GM-CSF-induced CD11c^+^CD8a^−^ dendritic cells facilitate Foxp3^+^ and IL-10^+^ regulatory T cell expansion resulting in suppression of autoimmune thyroiditis. Int. Immunol..

[37] Saas P, Perruche S (2012). Functions of TGF-β-exposed plasmacytoid dendritic cells. Crit. Rev. Immunol..

[38] Layseca-Espinosa E (2013). CCL22-producing CD8α^−^ myeloid dendritic cells mediate regulatory T cell recruitment in response to G-CSF treatment. J. Immunol..

[39] Tsang, M. L. S., Weatherbee, J. A., Dietz, M., Kitamura, T. & Lucas, R. C. TGF-beta specifically inhibits the IL-4 dependant proliferation of multifactor-dependent murine T-helper and human hematopoietic cell lines. *Lymphokine. Res.* **9**, 607–609 (1990).

[40] Pitha PM (1998). Role of the interferon regulatory factors (IRFs) in virus-mediated signaling and regulation of cell growth. Biochimie.

[41] Marié I, Durbin JE, Levy DE (1998). Differential viral induction of distinct interferon-alpha genes by positive feedback through interferon regulatory factor-7. EMBO J..

[42] Salio M, Palmowski MJ, Atzberger A, Hermans IF, Cerundolo V (2004). CpG-matured murine plasmacytoid dendritic cells are capable of in vivo priming of functional CD8 T cell responses to endogenous but not exogenous antigens. J. Exp. Med..

[43] Hunter CA (2005). New IL-12-family members: IL-23 and IL-27, cytokines with divergent functions. Nat. Rev. Immunol..

[44] Fitzgerald DC (2007). Suppressive effect of IL-27 on encephalitogenic Th17 cells and the effector phase of experimental autoimmune encephalomyelitis. J. Immunol..

[45] Pallotta MT (2011). Indoleamine 2,3-dioxygenase is a signaling protein in long-term tolerance by dendritic cells. Nat. Immunol..

[46] Diana J (2011). Viral infection prevents diabetes by inducing regulatory T cells through NKT cell-plasmacytoid dendritic cell interplay. J. Exp. Med..

[47] Sisirak V (2013). Breast cancer-derived transforming growth factor-β and tumor necrosis factor-α compromise interferon-α production by tumor-associated plasmacytoid dendritic cells. Int. J. Cancer.

[48] El-Behi M (2011). The encephalitogenicity of T(H)17 cells is dependent on IL-1- and IL-23-induced production of the cytokine GM-CSF. Nat. Immunol..

[49] Codarri L (2011). RORγt drives production of the cytokine GM-CSF in helper T cells, which is essential for the effector phase of autoimmune neuroinflammation. Nat. Immunol..

[50] Menon M, Blair PA, Isenberg DA, Mauri C (2016). A regulatory feedback between plasmacytoid dendritic cells and regulatory B cells is aberrant in systemic lupus erythematosus. Immunity.

[51] Bauer M (2001). Bacterial CpG-DNA triggers activation and maturation of human CD11c^−^, CD123^+^ dendritic cells. J. Immunol..

[52] Mancardi G, Sormani MP, Muraro PA, Boffa G, Saccardi R (2017). Intense immunosuppression followed by autologous haematopoietic stem cell transplantation as a therapeutic strategy in aggressive forms of multiple sclerosis. Mult. Scler..

[53] Muraro PA (2017). Autologous haematopoietic stem cell transplantation for treatment of multiple sclerosis. Nat. Rev. Neurol..

[54] Lutterotti A (2018). Challenges and needs in experimental therapies for multiple sclerosis. Curr. Opin. Neurol..

[55] Massey JC, Sutton IJ, Ma DDF, Moore JJ (2018). Regenerating immunotolerance in multiple sclerosis with autologous hematopoietic stem cell transplant. Front. Immunol..

[56] Mariottini A, De Matteis E, Muraro PA (2020). Haematopoietic stem cell transplantation for multiple sclerosis: Current status. BioDrugs.

