# Supplementary material for: Toll-like receptor-9 stimulated plasmacytoid dendritic cell precursors suppress autoimmune neuroinflammation in a murine model of multiple sclerosis
Source: Sci Rep. 2021 Feb 26;11:4735. doi: 10.1038/s41598-021-84023-0 (PMC7910458; doi:10.1038/s41598-021-84023-0)
Supplement: Supplementary file 1 — Supplementary Information. [file 41598_2021_84023_MOESM1_ESM.pdf]

Toll-like receptor-9 stimulated plasmacytoid dendritic cell precursors suppress autoimmune neuroinflammation in a murine model of multiple sclerosis

Hélène LETSCHER*, Viviane A. AGBOGAN*, Sarantis KORNIOTIS,
Pauline GASTINEAU, Emmanuel TEJERINA, Christophe GRAS,
Jérôme MEGRET, Alison MOE, William R. DROBYSKI and Flora ZAVALA

Supplementary material

Supplementary Figure S1

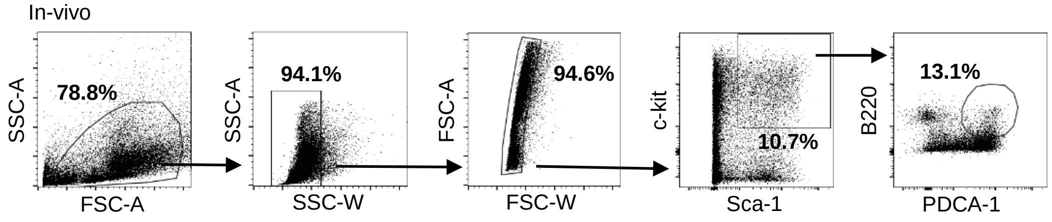

Figure S1: FACS cell sorting procedure of c-kit^{+}Sca-1^{+}B220^{int}PDCA-1^{+} cells recovered from c-kit^{+} cells magnetically selected from the BM of mice 18h after i.p. injection with 30 μg/ml CpG-B.

Supplementary Figure S2

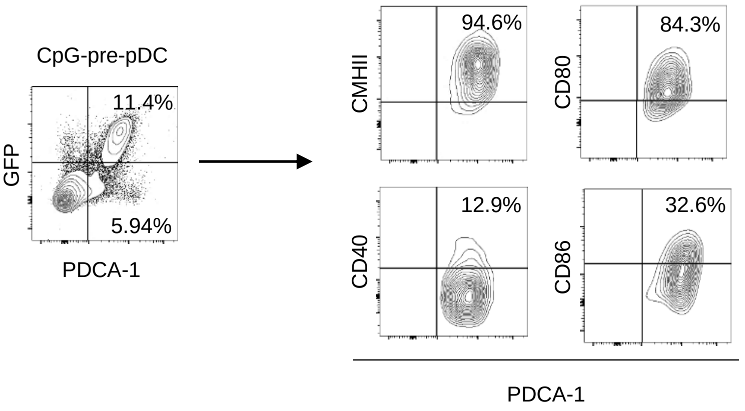

Figure S2: MHC-II and co-stimulatory markers expression in GFP^{+} CpG-pre-pDCs, adoptively transferred at d-12 and recovered at d-18 as GFP^{+} PDCA-1^{+} cells from the spinal cord of EAE recipients.

Supplementary Figure S3

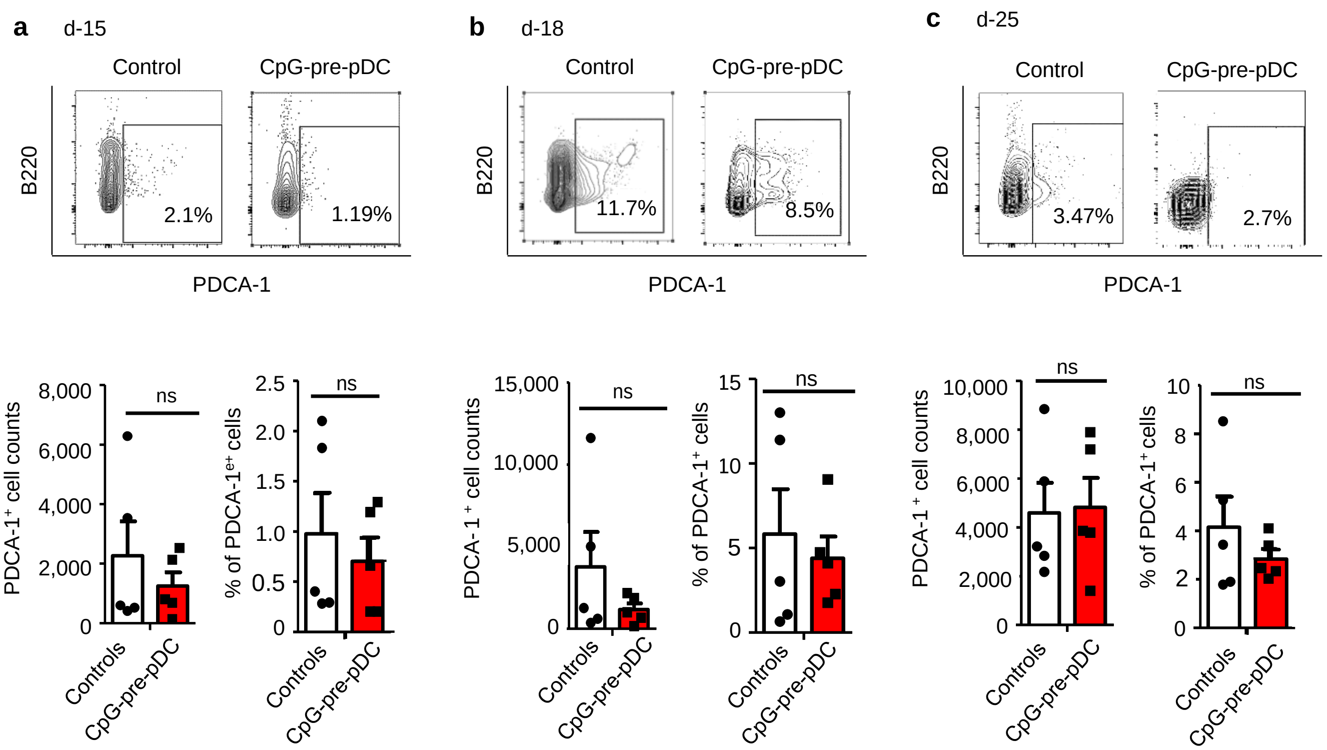

Figure S3: FACS analysis of B220⁺ PDCA-1⁺ cells in the spinal cord of mice immunized with EAE, either control or recipient of CpG-pre-pDCs adoptively transferred at d-12. Analysis was performed at day-15, day-18 and day-25 after immunization.

Supplementary Figure S4

a

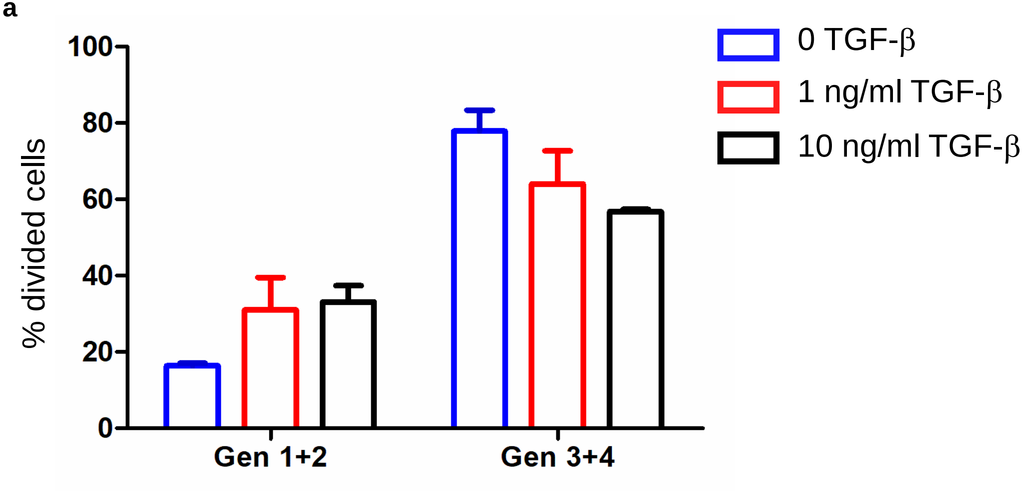

b

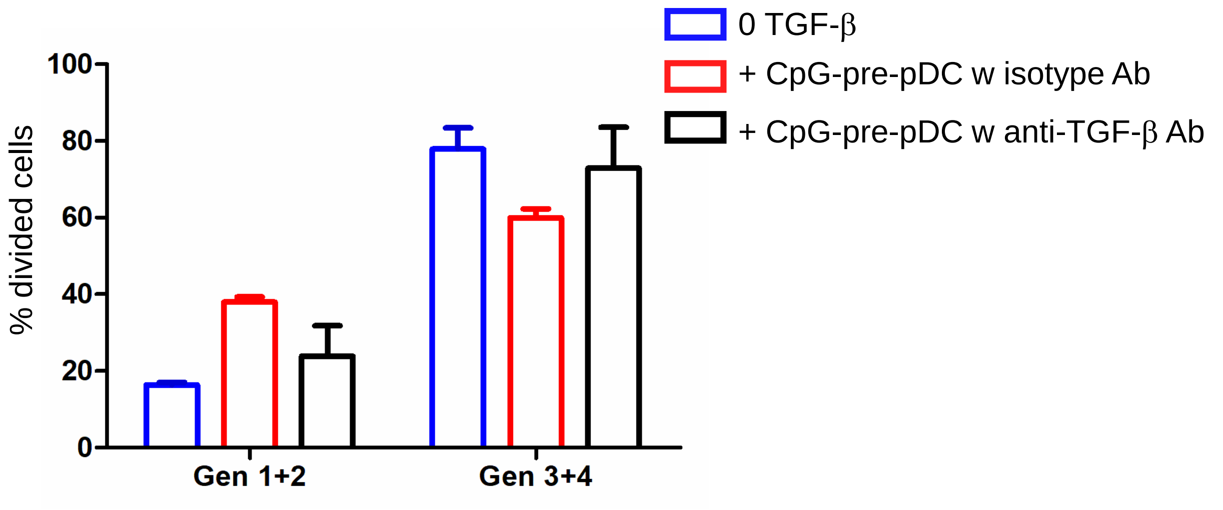

Figure S4: Reduced biodetection of TGF-β in lysates of CpG-pre-pDCs transfected with a neutralizing anti-TGF-β antibody: HT-2 cells were loaded with Cell Trace and stimulated with 7.5 ng/ml IL-4 for 48h **(a) TGF-β dose-dependent inhibition of the IL-4 dependent proliferation of the HT-2 cells.** Incubation with 0, 1 or 10 ng/ml recombinant TGF-β. Inhibition of HT-2 proliferation is translated by enhanced cell proportions in generations 1+2 of divided cells and reduced proportions of cells in advanced generations 3+4. 2 cumulated experiments. **(b) Neutralization of TGF-β bioactivity in anti-TGF-β transfected pDC progenitors.** Incubation of HT-2 cells with or without (blue histograms) lysates of 50,000 cell-sorted CpG-pre-pDCs transfected with the Chariot vector coupled to an isotype Ab (red histograms) or to a TGF-β neutralizing Ab (black histograms). Inhibition of HT-2 proliferation translated by enhanced generations 1+2 of cells in division show that the lysates of isotype Ab transfected progenitors (red histograms) contain TGF-β bioactivity while the lysates of anti-TGFβ transfected progenitors (black histograms) have reduced inhibitory activity, with percentages of cells in generations 1+2 close to levels observed in the absence of TGF-β (in blue histograms). 2 cumulated experiments.

Supplementary Figure S5

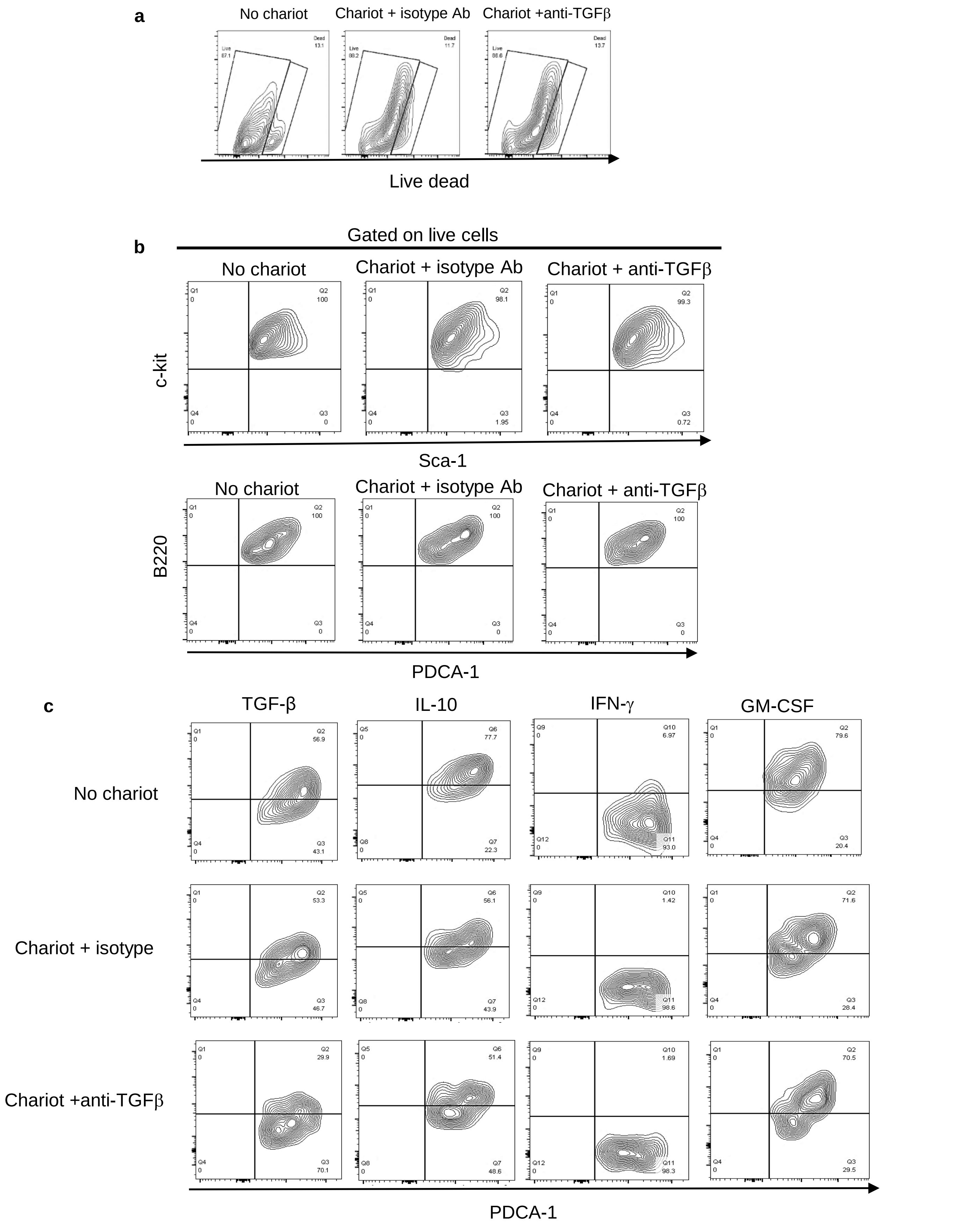

Figure S5: The Chariot transfection system does not alter the viability, phenotype and functional properties of CpG-pre-pDCs. Cell-sorted CpG-pre-pDCs were transfected (or not) with the Chariot vector complexed with either isotype Ab or anti-TGFβ. Cells were thereafter assessed for **(a)** Viability with Life dead staining, **(b)** Phenotype with c-kit, Sca-1, B220 and PDCA-1 staining and **(c)** Cytokine expression after 4h-activation with PMA + ionomycin + Golgi Stop.

Supplementary Figure S6

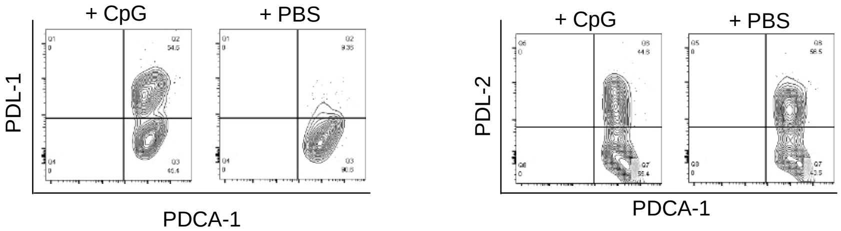

Figure S6: PDL-1 and PDL-2 expression, analyzed by flow cytometry, in cell-sorted CpG-versus PBS-pre-pDCs.
